# Intranasal vaccination with an NDV-vectored SARS-CoV-2 vaccine protects against Delta and Omicron challenges

**DOI:** 10.1038/s41541-024-00870-8

**Published:** 2024-05-23

**Authors:** Bryce M. Warner, Jacob G. E. Yates, Robert Vendramelli, Thang Truong, Courtney Meilleur, Lily Chan, Alexander Leacy, Phuc H. Pham, Yanlong Pei, Leonardo Susta, Sarah K. Wootton, Darwyn Kobasa

**Affiliations:** 1 Special Pathogens Program, National Microbiology Laboratory, Public Health Agency of Canada, Winnipeg, Canada; 2 Department of Pathobiology, University of Guelph, Guelph, N1G 2W1 Canada; 3 Department of Medical Microbiology and Infectious Diseases, University of Manitoba, Winnipeg, Canada

**Keywords:** Live attenuated vaccines, SARS-CoV-2

## Abstract

The rapid development and deployment of vaccines following the emergence of severe acute respiratory syndrome coronavirus 2 (SARS-CoV-2) has been estimated to have saved millions of lives. Despite their immense success, there remains a need for next-generation vaccination approaches for SARS-CoV-2 and future emerging coronaviruses and other respiratory viruses. Here we utilized a Newcastle Disease virus (NDV) vectored vaccine expressing the ancestral SARS-CoV-2 spike protein in a pre-fusion stabilized chimeric conformation (NDV-PFS). When delivered intranasally, NDV-PFS protected both Syrian hamsters and K18 mice against Delta and Omicron SARS-CoV-2 variants of concern. Additionally, intranasal vaccination induced robust, durable protection that was extended to 6 months post-vaccination. Overall, our data provide evidence that NDV-vectored vaccines represent a viable next-generation mucosal vaccination approach.

## Introduction

Severe acute respiratory syndrome coronavirus 2 (SARS-CoV-2) is the third zoonotic betacoronavirus to have emerged in the last two decades^1^. In contrast to its predecessors, severe acute respiratory syndrome coronavirus (SARS-CoV) and Middle East respiratory syndrome coronavirus (MERS-CoV), SARS-CoV-2 is the first to result in a global pandemic, termed coronavirus disease 2019 (COVID-19)^2^. COVID-19 is characterized as a respiratory illness, with disease outcomes ranging from mild symptoms to severe respiratory disease and death^2^. Increased disease severity has been linked to infection of the lower respiratory tract and is more often observed in individuals with pre-existing health conditions, immunodeficiencies, or old age^2,3^. The majority of SARS-CoV-2-infected individuals experience mild to moderate respiratory illness and recover without requiring special treatment, in part due to the rapid onset of spike glycoprotein-specific immune responses and confinement of infection to the upper respiratory tract^3–5^. This highlights the importance of mucosal immune responses in limiting infection and disease severity^5–7^.

The rapid spread of SARS-CoV-2 in comparison to other coronaviruses is attributed to alterations in the spike protein, such as a furin cleavage site, which allows for cleavage of the spike protein during virion assembly and budding, leading to increased infection efficiency^2,8^. The role of the spike protein in mediating viral attachment and entry, in addition to previous research on the development of SARS-CoV and MERS-CoV vaccines, led to its use as the target antigen in SARS-CoV-2 vaccine development^9–11^. Of the several COVID-19 vaccines that have received regulatory approval, two mRNA vaccines expressing the spike protein are predominantly used; the first being the BNT162b2 manufactured by BioNTech-Pfizer, and the second mRNA-1273 manufactured by Moderna-NIAID^12^. Following intramuscular administration of mRNA vaccines, potent systemic humoral and cell-mediated immunity against spike is observed, which protects against severe COVID-19^13–17^. The current method of parenteral mRNA vaccination induces systemic spike-specific B and T cell immunity but falls short in its ability to induce mucosal immune responses, indicated by their absence in the bronchoalveolar fluid of mRNA-vaccinated individuals^7,16–24^. The emergence of VOCs and the infections of vaccinated individuals has expanded how current vaccination strategies can be evaluated. Individuals who experience infection prior to or after mRNA vaccination exhibit more robust mucosal immune responses than only vaccinated individuals^18,19,24,25^.

Mucosal vaccines, typically administered intranasally, provide the benefit of inducing robust protection to the respiratory tract as well as systemically, which may be more effective at reducing SARS-CoV-2 infection and transmission^26,27^. Indeed, several intranasally administered SARS-CoV-2 vaccine candidates are currently being evaluated^7,28,29^, including recombinant Newcastle disease virus, vectored vaccines (NDV) expressing the pre-fusion stabilized SARS-CoV-2 spike protein, both in replicating and inactivated formats (NDV-HXP-S)^30–34^. Encouragingly, interim results from the initial clinical trials have demonstrated that the vaccine is safe and immunogenic^32^. As a vaccine vector, NDV has numerous advantages, including a natural tropism for respiratory epithelial cells resulting in local and systemic immunity^35,36^, little to no pre-existing immunity to the vaccine vector, a strong safety profile in humans^37–39^, the ability to easily engineer and stably express foreign transgenes, and ease of production in embryonated chicken eggs, all of which support its development as a vaccine vector for COVID-19^28,40–43^.

Described within is NDV-PFS, a live NDV viral-vectored vaccine against COVID-19. The ancestral spike has been modified in two important ways: (1) stabilized in the pre-fusion conformation and (2) modified to use the NDV F protein transmembrane domain and cytoplasmic tail. These modifications may allow the spike protein to be displayed at higher quantities and in a conformation that is likely more advantageous for immune responses^44,45^. If this is the case, mucosal and systemic immune responses providing protection against two VOCs will be evident. Herein, when NDV-PFS is administered intranasally, its ability to induce mucosal and systemic immune responses and provide protection against Delta and Omicron variants in K18-hACE2 mice and Syrian hamsters is described. After one dose, durable systemic humoral and cell-mediated immune responses that protected animals from challenge with VOCs were observed. Following two doses, Syrian hamsters were protected from VOC infection six months post-vaccination, demonstrating the durability of immunity induced by this vaccine vector.

## Results

### Efficient spike protein expression and virion incorporation from recombinant NDV encoding a chimeric pre-fusion stabilized SARS-CoV-2 spike protein

**Fig. 1:**
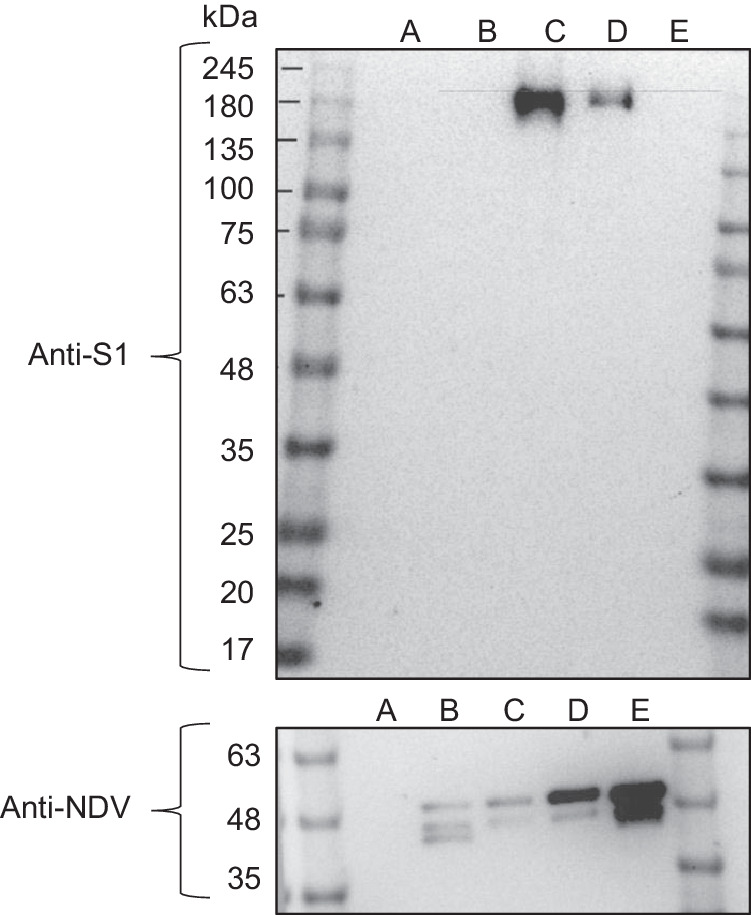
Western blot analysis of NDV-PFS. Western blot analysis of spike protein expression from uninfected (A), NDV-GFP (B) or NDV-PFS (C) infected cell lysates (100 μg of protein) and 5 × 10^4^ PFU of NDV-PFS (D) or NDV-GFP (E) purified viral stocks. Full-length spike protein (180 kDa) was detected using an anti-S1 antibody, with infection confirmed by the presence of NDV ribonucleoprotein (RNP) complex. DF1 cells were infected at an MOI of 1 for 24 h with either NDV-GFP, NDV-PFS, or left uninfected to generate cell lysates. Full-length spike protein expression is evident following NDV-PFS infection and on the surface of NDV-PFS compared to NDV-GFP, NDV-GFP-infected cells, and uninfected cells. The blots shown were processed in parallel for the detection of either SARS-CoV-2 or NDV.

In our previous work, the immunogenicity and efficacy of an NDV vaccine expressing the ancestral SARS-CoV-2 spike protein were highlighted^28^. However, following its characterization, the spike could not be detected in its pre-fusion conformation on the surface of NDV (Supplementary Fig. 1)^28^. Given the improved efficacy of pre-fusion stabilized spike proteins as vaccine antigens, this prompted the engineering of NDV-PFS, an NDV-expressing SARS-CoV-2 spike protein with the HexaPro proline substitutions^45^. In addition to an ablated furin cleavage site, pre-fusion stabilized spike protein of ancestral SARS-CoV-2 was further modified by substitution of the endogenous transmembrane and cytoplasmic domains for those of the NDV F protein, yielding a chimeric pre-fusion stabilized spike protein. Western blot analysis of spike protein expression from NDV-PFS infected cell lysates revealed a single band of ~180 kDa in size representing an uncleaved spike (Fig. 1). A similar-sized molecular weight band was detected in the lane loaded with 5 × 10^4^ PFU of purified in vivo grade NDV-PFS indicating that the chimeric pre-fusion stabilized spike protein was efficiently incorporated into the NDV virion (Fig. 1). These findings supported further characterization of NDV-PFS as a vaccine for SARS-CoV-2.

### Intranasal vaccination with NDV-PFS provides protection against Delta and Omicron variants of concern in Syrian hamsters

**Fig. 2:**
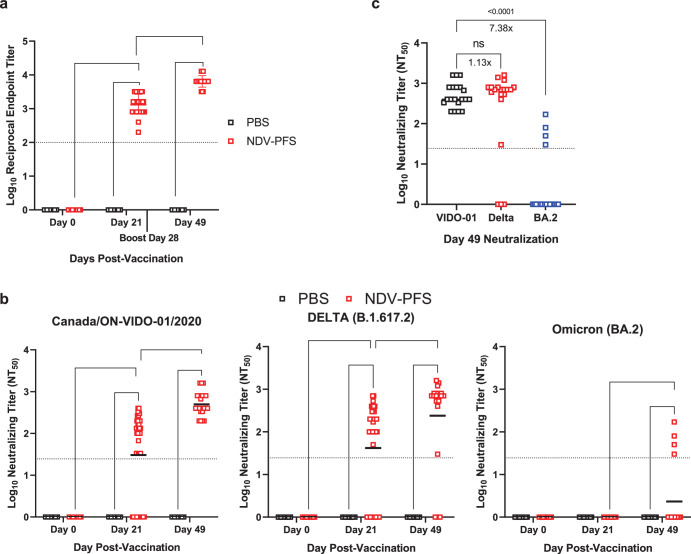
Humoral immune responses following NDV-PFS vaccination in hamsters. Syrian hamsters were vaccinated intranasally with NDV-PFS. **a** WT SARS-CoV-2 spike protein-specific serum IgG endpoint titers 21 days following each vaccine dose. **b** WT SARS-CoV-2, Delta, and Omicron (BA.2) neutralization titers 21 days following each vaccine dose. **c** Neutralization titers against WT SARS-CoV-2, Delta, and Omicron (BA.2) 21 days following the second vaccine dose, with fold differences in mean titers shown. Reported are Log_10_ NT_50_ values. Significance was assessed using two-way ANOVA in (**b**) and Kruskal–Wallis test in (**c**). ***p = 0.004; ****p < 0.0001. *n* = 40 for day 21 and *n* = 20 for samples on day 49. Shown are geometric mean titers + SD and **a** and **b**, medians are shown in (**c**).

We previously showed that vaccination of Syrian hamsters via the intranasal route with NDV expressing ancestral SARS-CoV-2 spike protein was immunogenic and provided strong protection from infectious challenges after two doses^28^. Here, we examined the immunogenicity and protective efficacy of the pre-fusion stabilized formulation of the NDV vaccine in hamsters in a similar manner. Intranasal vaccination with 1 × 10^7^ PFU of NDV-PFS resulted in robust ancestral spike-specific IgG titers in the serum of hamsters three weeks following the first and second doses of vaccine (Fig. 2a). Following the booster dose, a significant increase in IgG was detectable in the serum of vaccinated hamsters, indicating a boost effect. Neutralization of ancestral SARS-CoV-2 was also seen in the majority of vaccinated hamsters 21 days following the first vaccine dose and then in 100% of vaccinated animals 21 days following the second dose (Fig. 2b). Because the vaccinated animals in this experiment were to be challenged with SARS-CoV-2 variants of concern Delta (B.1.617.2) and Omicron (BA.2), we also performed neutralization assays against both variant viruses. Neutralizing titers against Delta were detectable in 28/40 animals following a single dose of NDV-PFS and were significantly higher than in control animals (Fig. 2b). Following the booster dose in 20 animals, all but three had measurable neutralizing antibody responses, and the mean neutralization titer had increased significantly over that seen following only a single dose when examined three weeks after the booster (Fig. 2b). For BA.2, only 4 animals that had received both doses had detectable neutralizing antibodies, confirming the immune evasive properties of this lineage of SARS-CoV-2 (Fig. 2b). No animals had BA.2-neutralizing antibody responses following the first dose. Comparing mean neutralization titers against all three viruses, neutralization of ancestral SARS-CoV-2 was highest; however, only 1.13-fold higher than the mean neutralization titers against Delta, and this difference was not statistically significant (Fig. 2c). The mean neutralization titer seen against ancestral SARS-CoV-2 was more than 7-fold higher than that seen against BA.2, again confirming the significant humoral immune evasion of Omicron.

**Fig. 3:**
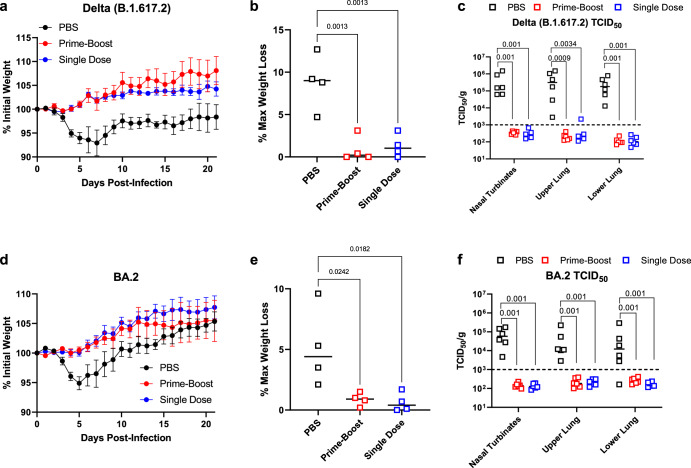
Protective efficacy of NDV-PFS vaccination against SARS-CoV-2 variants of concern. Syrian hamsters were vaccinated with NDV-PFS and challenged 28 days following their final dose with either Delta in (**a**–**c**) or Omicron (BA.2) in (**d**–**f**). **a** Weight loss in Delta-challenged hamsters. **b** Maximum weight loss in each of the individual hamsters challenged with Delta that were monitored throughout the course of the experiment. **c** TCID_50_ titers in the tissues of each Delta-infected animal euthanized on day 5 post-infection. **d** Weight loss in BA.2 challenged hamsters. **e** Maximum weight loss in each of the individual hamsters challenged with BA.2 that were monitored throughout the course of the experiment. **f** TCID_50_ titers in the tissues of each BA.2 infected animals were euthanized on day 5 post-infection. In **b**, **c**, **e**, and **f**, significance was assessed by the Kruskal–Wallis test with multiple comparisons. Exact *p* values are shown where significant differences are seen. *n* = 10 in **a** and **d** until day 5, and then *n* = 4. *n* = 4 in (**b** and **e**). *n* = 6 in (**c** and **f**). Shown are means + SEM in (**a** and **d**) and medians in (**b**, **c**, **e**, **f**).

We challenged single-dose and prime-boost vaccinated hamsters with 10^5^ TCID_50_ of either SARS-CoV-2 Delta or Omicron (BA.2). Regardless of whether animals received one or two doses of NDV-PFS, significant protection from weight loss was seen following Delta infection (Fig. 3a). The mean maximum weight loss seen in the four animals examined past day 5 showed a significant difference between both vaccine groups and PBS controls (Fig. 3b). We also euthanized and necropsied six animals from each group on day five post-infection to examine viral titers in respiratory tissues. All control animals had detectable infectious virus in the nasal turbinates and lung sections tested. Median titers above 10^5^ TCID_50_ per gram were seen in all tissues (Fig. 3c). In contrast, all animals that received two doses of vaccine had no detectable infectious virus. Additionally, only a single upper lung sample from a single dose vaccinated animal had detectable infectious virus, indicating that either one or two doses provide significant protection from viral replication in the upper and lower airways in Syrian hamsters infected with Delta. Following BA.2 infection, clinical disease in Syrian hamsters is not as severe as it is following infection with ancestral SARS-CoV-2 or the Delta variant^46^. However, we did see modest weight loss, peaking at about 5% on day 5 after infection in most control BA.2 infected hamsters, with only one animal losing just under 10% of its initial starting weight (Fig. 3d, e). Vaccination with one or two doses of NDV-PFS again provided significant protection from weight loss compared with the control group, with vaccinated animals experiencing no weight loss (Fig. 3d). The maximum weight loss seen in each group among the four animals examined beyond day five again showed significant reductions in weight loss between the vaccine groups and the controls (Fig. 3e). Following BA.2 challenge, again six animals were euthanized on day five post-infection and tissues were collected for determination of viral titers. While median viral titers in the nasal turbinates and lungs were lower following BA.2 challenge in the control group compared with Delta infection, once again significant reductions were seen in viral titers in both vaccine groups (Fig. 3f). No infectious virus was detected in any of the tissue samples from either vaccine group, indicating strong protection in the airway against BA.2 infection, despite its antigenic divergence from ancestral SARS-CoV-2. We were able to detect SARS-CoV-2 spike-specific IgA in lung homogenates from animals taken on day 5 in both vaccine groups (Supplementary Fig. 2). We did not detect a signal in the lung homogenates of animals in the PBS group, suggesting that intranasal vaccination did induce IgA production, which may have played a role in protection in vaccinated hamsters. In addition, we assayed BA.2 neutralization using post-challenge serum taken on day 5 in protected animals and in controls. Interestingly, in both the single dose and prime-boost groups, a significant increase in BA.2-neutralizing antibodies was seen compared with pre-challenge serum (Supplementary Fig. 3A). This suggests that undetectable anti-BA.2 or cross-reactive neutralizing antibodies were present prior to infection and that BA.2 infection boosted the production of these antibodies by memory B cells. Histopathological evaluation of the lungs showed that vaccinated hamsters challenged with either Delta or Omicron exhibited a reduction in lung pathology score relative to the control group, also when hamsters were challenged 186 days after boost (Supplementary Fig. 4A, B). While significant most of the time, these differences were not when comparing control vs. prime-boost vaccine groups infected with the Delta (at 5 days) and Omicron (at 186 days), most likely due to the high dispersal of severity scores in the control groups. Most control hamsters exhibited exudative lesions, consisting of an alveolar accumulation of fibrin/edema, sloughed cells, and macrophages, with variable interstitial pneumonia. In vaccinated hamsters, exudative lesions were minimal, and epithelial hyperplasia could be observed (repair) (S5A-H). Overall, our data indicate that one or two intranasal doses of NDV-PFS can provide robust protection against disease and viral replication following infection with two distinct SARS-CoV-2 variants of concern.

### Intranasal vaccination with NDV-PFS protects against Delta and Omicron variants of concern in K18 transgenic mice

**Fig. 4:**
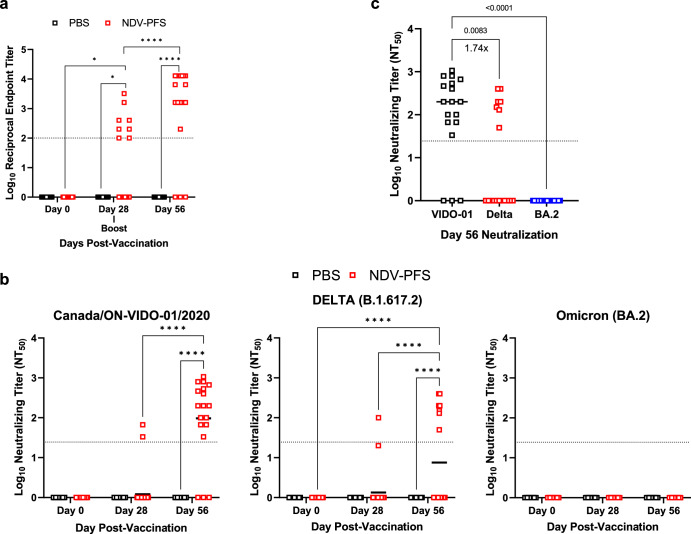
Humoral immune responses following NDV-PFS vaccination in K18 mice. K18 transgenic were vaccinated intranasally with NDV-PFS. **a** WT SARS-CoV-2 spike protein-specific serum IgG endpoint titers in 28 days following each vaccine dose. **b** WT SARS-CoV-2, Delta, and Omicron (BA.2) neutralization titers 28 days following each vaccine dose. **C** Neutralization titers against WT SARS-CoV-2, Delta, and Omicron (BA.2) 28 days following the second vaccine dose, with fold differences in mean titer between WT and Delta shown. Reported are Log_10_ NT_50_ values. Significance was assessed using two-way ANOVA in **b** and the Kruskal–Wallis test in (**c**). ***p = 0.004; ****p < 0.0001. *n* = 40 for day 21 and *n* = 20 for samples on day 56. Shown are geometric mean titers + SD and **a** and **b**, medians are shown in (**c**).

While we were able to show protection against both Delta and BA.2 challenges following vaccination in Syrian hamsters, we wanted to corroborate these results by assessing protection afforded by NDV-PFS in a second animal model of SARS-CoV-2 infection. The K18 human ACE2 transgenic mouse model is a well-characterized model of SARS-CoV-2 infection and represents a lethal model following infection with ancestral SARS-CoV-2 and certain variants due to neuro-invasion of the virus and lethal encephalopathy^47,48^. Here, we utilized the K18 mouse model to determine whether NDV-PFS protects a second animal species, using both a lethal Delta challenge and a non-lethal BA.2 challenge. In this case, animals received one or two doses of 1 × 10^6^ PFU of NDV-PFS intranasally, with serum collections performed 4 weeks following the first or second dose of the vaccine. Surprisingly, only 7 out of 36 animals had detectable ancestral (WT) SARS-CoV-2 spike-specific IgG titers in the serum after a single dose of vaccine (Fig. 4a). After the booster dose, of the 18 animals that received a second dose, all but three had detectable IgG titers, indicating that a second vaccine dose was necessary for seroconversion in most cases with the vaccine administered to mice via the intranasal route (Fig. 4a). We measured neutralizing antibody responses in vaccinated mice against ancestral SARS-CoV-2, Delta, and BA.2 as we did above in hamsters. After one dose, only two animals had neutralizing antibodies against the ancestral virus, however after a second dose, all but three animals had detectable neutralizaing antibodies (Fig. 4b). A similar trend was seen against Delta, with only a single animal having neutralizing antibodies above the limit of detection after one dose, however just seven of the 18 boosted animals had some level of neutralizing antibodies following the second dose (Fig. 4b). Against BA.2, no neutralizing antibodies were detected in any of the vaccinated mice, even after two doses (Fig. 4b). Comparing mean neutralization titers against ancestral SARS-CoV-2 and Delta, there was a 1.74-fold reduction in mean titer, however more than half of boosted mice did not have detectable Delta-neutralizing antibodies (Fig. 4c). This absent to modest neutralizing response seen in mice indicates the NDV platform may not be as immunogenic in mice compared with hamsters, at least when given via a mucosal intranasal inhalation route.

**Fig. 5:**
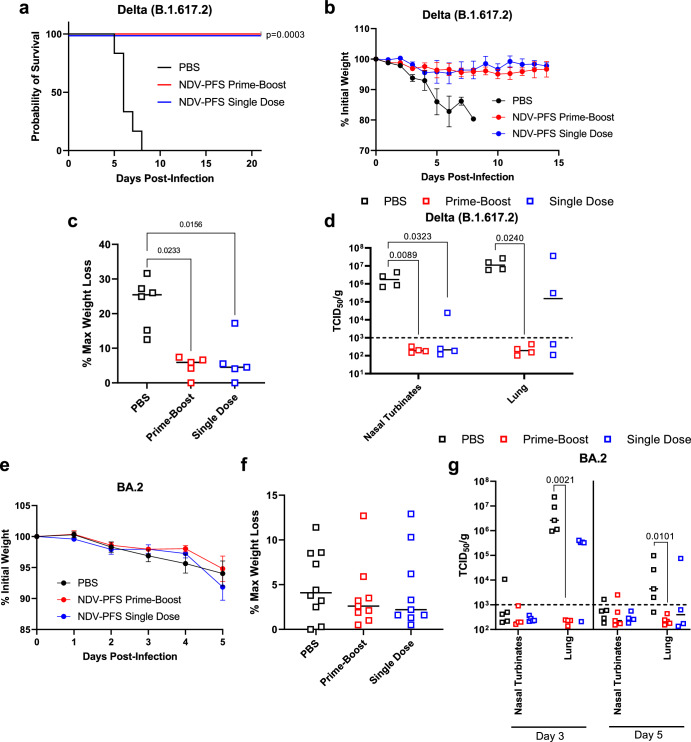
Protective efficacy of NDV-PFS vaccination against SARS-CoV-2 variants of concern in mice. K18 mice were vaccinated with NDV-PFS and challenged 28 days following their final dose with either Delta in (**a**–**d**) or Omicron (BA.2) in (**e**–**g**). **a** Survival of K18 mice following Delta challenge. **b** Weight loss in Delta-challenged hamsters. **c** Maximum weight loss in each of the individual hamsters challenged with Delta that were monitored throughout the course of the experiment. **d** TCID50 titers in the tissues of each Delta-infected animal euthanized on day 3 post-infection. **e** Weight loss in BA.2 challenged hamsters. **f** Maximum weight loss in each of the individual hamsters challenged with BA.2 that were monitored throughout the course of the experiment. **g** TCID50 titers in the tissues of each BA.2 infected animals euthanized on days 3 or 5 post-infection. In **c**, **d**, and **g**, significance was assessed by one-way ANOVA with multiple comparisons. Exact *p* values are shown where significant differences are seen. *n* = 5 or 6 in (**a**). *n* = 10 in (**b**) until day 3, and then *n* = 6. *n* = 5 or 6 in (**c**). *n* = 4 in (**d**). *n* = 10 in (**e**) until day 5, and *n* = 5 until day 5. *n* = 10 in (**f**). *n* = 5 per time point in (**g**). Shown are means + SEM in (**b**) and (**e**) and medians in (**c**), (**d**), (**f**), and (**g**).

Once again, we challenged control and vaccinated animals with either SARS-CoV-2 Delta or BA.2 variants. In the lethal Delta challenge model, all control animals succumbed to disease by day eight post-infection (Fig. 5a). Both single-dose and prime-boost groups saw 100% survival rates despite modest humoral immune responses, and even absent neutralizing antibodies in some animals (Fig. 5a). PBS control animals began to lose weight on day three post-infection, and rapidly deteriorated prior to euthanasia on days 6–8, while both vaccine groups had minor weight loss, but were protected from severe weight loss and disease development (Fig. 5b). The maximum weight loss seen in each group showed that control animals had severe weight loss requiring euthanasia, while animals in each vaccine group did experience weight loss but did not reach humane endpoint criteria (Fig. 5c). Interestingly, despite protection from severe weight loss, vaccinated animals infected with Delta did not gain weight following infection and mean weights in both vaccine groups remained at or just below their initial starting mean weights (Fig. 5b). We euthanized four animals from each group on day three during the acute phase of infection, to examine viral titers in the upper and lower airways. In the prime-boost group, no infectious virus was detected in either the nasal turbinates or the lungs of infected animals (Fig. 5d). In the single dose group, one and two out of four animals had detectable infectious virus in the nasal turbinates and lungs, respectively (Fig. 5d). Median viral titers in the control group were greater than 10^6^ and 10^7^ TCID_50_ per gram in nasal turbinates and lungs, while titers were significantly lower in the vaccinated groups, except for the lungs of the single-dose animals. Because BA.2 infection of K18 mice is not lethal, we measured weight loss for five days following infection and performed necropsies on days 3 and 5 to examine viral titers in the tissues. No differences in weight loss were seen after BA.2 challenge with all groups showing modest weight loss by day 5 (Fig. 5e). Maximum weight loss across all three groups was similar, with most animals that were kept until day 5 showing peak weight loss on that day (Fig. 5f). On days 3 and 5 post-infection, viral titers were undetectable in most nasal turbinate samples (including controls), indicating that infection was occurring mostly in the lower airways (Fig. 5g). In the lungs, only animals receiving two doses of vaccine had consistent reductions in viral titer compared with controls. In contrast to control animals that showed significant viral replication at day three and lower, but still significant, titers in most animals on day 5 were significantly lower than controls, even on day 5 when control titers were lower than on day three (Fig. 5g). Infectious virus was seen in three of four single-dose animal lung samples on day 3, with no significant difference between this group and controls. While three of four samples were below the limit of detection on day 5, the group average was not significantly different compared with the control group. Unlike hamsters, we did not observe serum neutralizing activity against BA.2 (Supplementary Fig. 3), a finding that is also consistent with the lack of detectable IgA in lung homogenates of infected mice (Supplementary Fig. 6), which is consistent with the low magnitude of humoral immune response generated in mice.

Pathological evaluation of lung tissues harvested on D3 and D5 post-challenge with Delta and BA.2, respectively, revealed no significant differences in the lesion scores of vaccinated and unvaccinated mice (Supplementary Fig. 4C). However, lesions were different from what was observed in hamsters, and were dominated by interstitial pneumonia with development of perivascular cuffs of lymphocytes with little to no exudative lesions in the alveoli (Supplementary Fig. 5I–P). This lymphocytic accumulation could explain decreased virus titers despite the lack of serum-neutralizing activity and support a cell-mediated response (see below). Overall, our data suggest that despite modest humoral immunogenicity in K18 mice and the absence of detectable neutralizing antibodies, intranasal vaccination with NDV-PFS can provide partial protection against infectious challenges.

### Intranasal vaccination with NDV-PFS induces cell-mediated immune responses in K18 transgenic mice

**Fig. 6:**
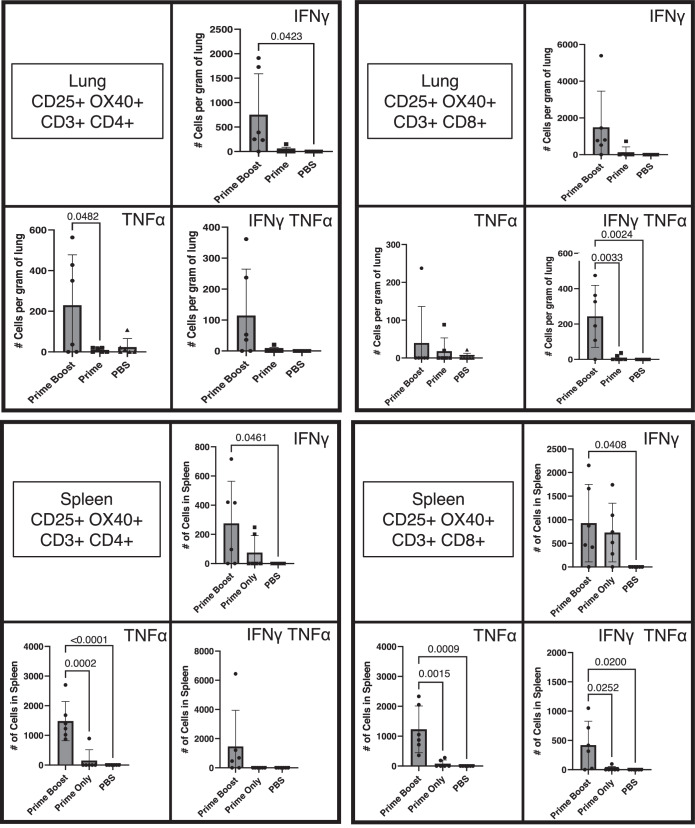
Cell-mediated immune response to NDV-PFS in K18 mice. Equal numbers of male and female K18 mice were not vaccinated (*n* = 6) or vaccinated with one (*n* = 6) or two (*n* = 6) doses of NDV-PFS and spleens and lungs were harvested 10 days or 5 days post-administration, respectively. Isolated cells were stimulated with ancestral spike peptides for 18 h prior to staining. Following the identification of live activated T cells (CD25+, OX40+), populations were further separated into CD4 (left panels) and CD8 T cells (right panels) (CD25+, OX40+, CD3+ CD4/CD8+) and assessed for polyfunctionality (IFNγ+ and TNFα+) in the lung (top panels) and spleen (bottom panels). Shown are mean values + SD. Significance was assessed by one-way analysis of variance and Tukey’s multiple comparisons test. Exact *p* values are shown where significant differences were seen.

Neutralizing antibodies have been suggested as a correlate of protection against COVID-19 disease development and infection with SARS-CoV-2^49–51^. Interestingly, in our mouse studies, we showed strong protection against weight loss, disease, and viral replication in a lethal Delta challenge model, even in animals receiving a single dose that did not have measurable neutralizing antibodies. We also noted partial protection against viral replication in a BA.2 challenge model in the complete absence of detectable neutralizing antibodies, even in animals receiving two doses. These data suggest that cell-mediated immune responses can contribute to NDV-PFS-mediated protection in the K18 mouse model. Therefore, we vaccinated K18 mice with NDV-PFS intranasally in an attempt to characterize the systemic and tissue-resident SARS-CoV-2-specific T-cell response. K18 mice received either one or two doses, as described in the “Methods” section. Compared to unvaccinated mice, two intranasal doses of NDV-PFS generated a significant amount of polyfunctional CD8+ T-cells producing both TNFα and IFNγ in the lungs and spleen (Fig. 6). Polyfunctional cells were also detected in mice receiving one dose, but to a lesser extent (Fig. 6). The generation of polyfunctional CD4+ T-cells in response to NDV-PFS vaccination appeared to follow a similar trend, albeit with greater variability (Fig. 6). All cells identified as polyfunctional also expressed the T-cell activation marker OX40, which was another marker used to show T cell activation^52^. Overall, our results indicate that intranasal vaccination of mice with NDV-PFS induced a SARS-CoV-2 spike-specific T cell response in both the spleen and lungs, which may be critical for protection from infection and disease in the K18 mouse model.

### Intranasal vaccination provides long-term protection against Delta and Omicron variants of concern in Syrian hamsters

**Fig. 7:**
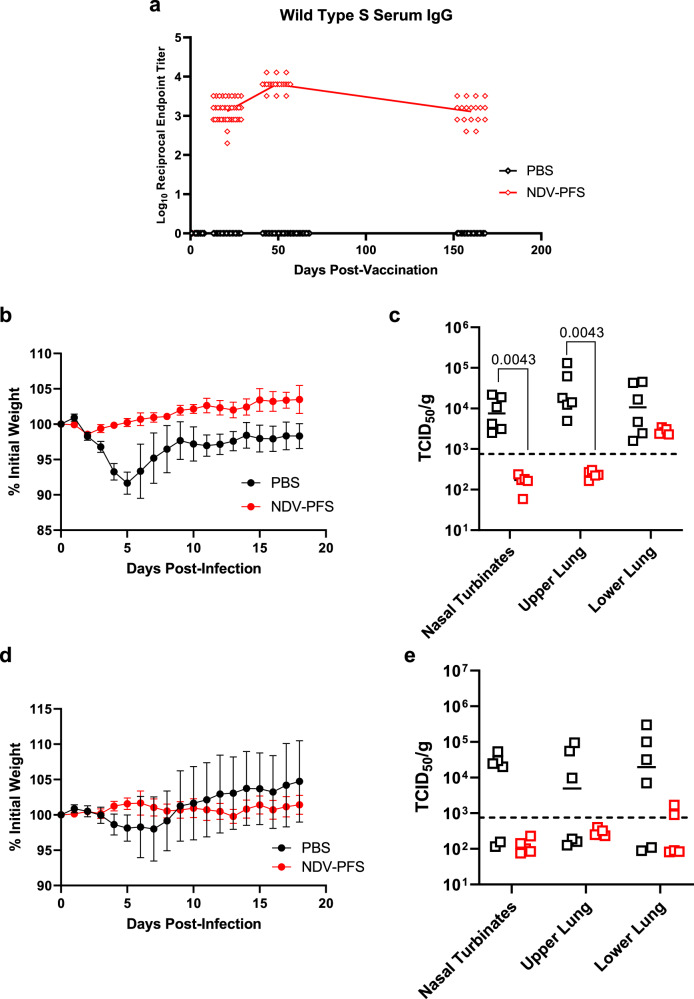
NDV-PFS-mediated protection of Syrian hamsters 168 days following vaccination. Syrian hamsters were vaccinated with NDV-PFS and challenged with either Delta in **b** and **c** or BA.2 in (**d**) and (**e**). **a** WT SARS-CoV-2 spike protein-specific serum IgG endpoint titers following vaccination. Endpoint titers at day 160 are shown along with the same data that is included in Fig. 2a. **b** Weight loss in Delta-challenged hamsters 168 days post-vaccination. **c** TCID50 titers in the tissues of each Delta-infected animal euthanized on day 5 post-infection. **d** Weight loss in BA.2 challenged hamsters. **e** TCID50 titers in the tissues of each BA.2 infected animals were euthanized on day 5 post-infection. In (**c**), significance was assessed by the Mann–Whitney test. Exact *p* values are shown where significant differences are seen. *n* = 18 at day 160 post-vaccination in (**a**). *n* = 9 or 10 in (**b**) and (**d**) until day 5, and then *n* = 4. *n* = 5 or 6 in (**c**) and (**e**). Shown are means in (**a**), mean + SEM in (**b**) and (**d**), and medians in (**c**) and (**e**).

Our infection experiment with Syrian hamsters indicated that intranasal vaccination with NDV-PFS provides strong protection against both Delta and BA.2 when the challenge is performed four weeks post-boost. One of the principal concerns regarding COVID-19 vaccinations is the durability of the immune response, with waning antibody levels leading to increased susceptibility to infection and disease sometime several months following vaccination^53–55^. Here, we aimed to determine whether our intranasal vaccination approach could provide durable, lasting protection against Delta and BA.2 challenge at 6 months post-vaccination, comparable to that seen at one-month post-vaccination. In Syrian hamsters that were vaccinated twice with NDV-PFS intranasally, SARS-CoV-2 spike-specific IgG in the serum remained at high levels at 160 days post-vaccination, though titers had fallen compared to those at 3 weeks post-boost, as expected (Fig. 7a). Day 160 serum titers were comparable to titers seen on day 21 following the initial dose. We challenged vaccinated hamsters with Delta or BA.2 on day 168 post-boost, along with unvaccinated age-matched control hamsters. Following the Delta challenge, control animals had moderate weight loss and did not return to their initial starting weight by day 18, the end of the study period (Fig. 7b). In contrast, NDV-PFS vaccinated animals had minor weight loss the first two days after infection followed by weight gain, showing significant protection from weight loss and clinical signs. On day five post-infection animals were necropsied for detection of viral titers in the nasal turbinates and the upper and lower lung. While control animals had high viral loads in all three tissue samples, vaccinated animals did not have detectable virus in the nasal turbinates or upper lungs (Fig. 7c). In the lower lung samples, all vaccinated animals had low levels of infectious virus present. Following the BA.2 challenge, weight loss was variable, with half of the infected control animals not showing any weight loss but weight gain following infection. Some animals did show moderate weight loss, in some cases up to 12% of their initial weight. This is reflected in the large standard error of the mean weights seen in Fig. 7d. Among vaccinated animals, no individual animal lost more than 2% of its initial weight, with most animals gaining weight following infection. On day 5 following the necropsy, viral titers in the tissues were examined. Interestingly, some animals in the control group did not have detectable infectious virus in their nasal turbinates or lungs (Fig. 7e). All tissue samples from vaccinated animals except for two lower lung samples were negative by TCID50, indicating that protection from viral replication remained in these animals out to 168 days following vaccination. Additionally, an absence of lung pathology in vaccinated hamsters was observed relative to unvaccinated hamsters (Supplementary Figs. 4 and 5). Overall, it appears as though intranasal vaccination with NDV-PFS provides substantial long-term protection against both Delta and BA.2 in Syrian hamsters.

## Discussion

We previously showed that intranasal vaccination with recombinant NDV expressing SARS-CoV-2 spike protein provides substantial protection from challenge in a Syrian hamster model of infection^28^. Our previous vaccine expressed the wild-type ancestral SARS-CoV-2 spike protein and protected in a homologous challenge model against the virus circulating globally at the time. Many of the spike proteins in commercial and approved COVID-19 vaccines feature the two-proline substituted pre-fusion stabilized version of the SARS-CoV-2 spike^10–12^. Therefore, we developed a new NDV vaccine expressing the pre-fusion stabilized spike featuring six proline substitutions, as described previously, and tested whether it could provide broad protection against two SARS-CoV-2 variants of concern in relevant animal models when given via intranasal vaccination^31,32^. Our results indicate that intranasal vaccination with NDV-PFS provides robust protection against Delta and BA.2 in both Syrian hamsters and K18 mice. The implications of these results are discussed here.

Engineering NDV to express a chimeric pre-fusion stabilized spike protein of SARS-CoV-2, with NDV F protein transmembrane and cytoplasmic domains, resulted in a recombinant NDV that incorporates the pre-fusion stabilized spike into its virion. In comparison to our previous work, less virus is required to detect more spike protein on the NDV virion^28^, suggesting greater incorporation of the pre-fusion spike into the NDV virion compared to wild-type spike lacking the NDV F protein transmembrane and cytoplasmic tail domains (Supplementary Fig. 1). An increased availability of antigen can support the induction of greater antibody responses, as seen in dose escalation studies with the Moderna COVID-19 mRNA vaccine^23,56^.

Initial emergency use authorizations for COVID-19 vaccines in North America occurred in late 2020-early 2021^57^. Since that time, despite high initial effectiveness against infection and symptomatic COVID-19, effectiveness against these parameters has dropped due to expected antibody waning and the continued evolution of SARS-CoV-2 toward new antibody-evading variants. While vaccine effectiveness remains high against hospitalization and severe disease, approaches that can induce immune responses at the mucosa where initial viral exposure occurs could protect against infection and transmission. Indeed, several intranasal and/or mucosal vaccination approaches have been developed and tested either pre-clinically or in early human trials^28,29,32,58–60^. We previously showed that intranasal vaccination using our first-generation NDV vaccine for SARS-CoV-2 provided strong protection in Syrian hamsters after one or two doses, and we revisited this approach using our PFS vaccine^28^. NDV-PFS vaccination was more immunogenic than the wild-type spike protein and induced high levels of spike-specific IgG^28^ (Fig. 2). As expected, serum neutralization titers against Delta and Omicron did not match titers against ancestral SARS-CoV-2, or titers seen previously, however, there was no significant reduction in mean neutralization titer against Delta compared with VIDO-01 in hamsters^61^. Strikingly, neutralization titers against BA.2 were non-existent after a single dose in hamsters and only detectable in four out of 20 animals after a booster dose. This contrasts both adenovirus and modified vaccinia Ankara (MVA) vaccines encoding the Wuhan spike in its wildtype or pre-fusion conformation (two proline substitution) where robust cross-reactive serum neutralizing antibodies are observed^62–67^. Induced antibodies exhibited varied binding abilities to different VOCs, as observed with the antibodies resultant of NDV-PFS vaccination^62,63,66,67^. Interestingly, one of the MVA vectored vaccines did not prevent SARS-CoV-2 (B.1) replication in the lungs^62^. Additionally, one of the adenovirus vaccines, but not the others, fell short in its ability to provide complete protection as well when administered intramuscularly, but this was avoided when administered intranasally^65^. Following two doses of NDV-HXP-S in phase I clinical trial (NCT04871737), serum-neutralizing antibodies were not readily identified in the intranasally vaccinated group unless an IM or IN and IM combined vaccination approach was used^68^. However, antibodies that inhibit the receptor binding domain interaction with the ACE2 receptor were present in all three groups tested, something which was not evaluated here^68^. Increases in activated CD4 and CD8 T cells isolated from peripheral blood mononuclear cells were also observed in the IN group, like what was observed above^68^. Interestingly, the IN/IM combination group elicited the greatest increases in activated CD4 and CD8 T-cells while maintaining antibody production, suggesting that a combination of intranasal and intramuscular vaccination routes would be best moving forward rather than only intranasal vaccination, used here^68^. Here, one or two doses of NDV-PFS provided strong protection against weight loss, viral replication, and lung pathology in hamster tissues, with similar outcomes observed in mice, aside from the presence of lung pathology (S8). Attempts at measuring serum IgA antibodies failed, thus we attempted to measure mucosal IgA in the lungs of infected animals following a planned necropsy. We did see an increased signal in an IgA ELISA specific for SARS-CoV-2 spike protein using lung homogenates from hamsters following infection (Supplementary Fig. 2). This signal was higher than that seen in the lungs of infected PBS-vaccinated animals in the lungs of uninfected control animals, or negative (PBS only) controls, indicating that there is spike-specific IgA present in the lungs following vaccination in hamsters; however, the same increase was not seen in mice (Supplementary Fig. 4). In addition to IgA, we examined the presence of BA.2 neutralizing antibodies following challenge to determine whether undetectable BA.2 neutralizing antibodies may have been present following vaccination. Significant increases in BA.2 neutralizing antibodies were seen in hamsters from both the single dose and prime-boost groups (Supplementary Fig. 3A) at 5 days post-challenge. Our data suggest that cross-reactive or BA.2-specific B cells are activated and primed following vaccination, and a robust BA.2-specific response is generated following the challenge, likely due to the engagement of memory B cells with BA.2 spike protein. This also suggests that pre-challenge serum antibody or neutralization levels may not necessarily indicate an individual’s susceptibility to infection or disease with a given variant, and attempts to identify memory B cell subsets or variant-specific T cells may be warranted to get a better understanding of possible protection. Our data are in line with recent studies showing that immune imprinting due to early exposures to the ancestral spike protein, either via vaccination or infection, can be overcome by exposure to Omicron spike proteins^69,70^. Due to limited immunological reagents available for examining cellular responses in hamsters, gaining insights into possible mechanisms of protection in vaccinated hamsters is difficult and cellular responses were not studied in hamsters here. However, we were able to show clear, robust protection from disease and viral replication in hamsters following mucosal vaccination. We should note that the intranasal route of vaccine administration used in this study for vaccination results in deposition of vaccine inoculum into the lungs in addition to the upper respiratory tract, resulting in generation of “respiratory immunity”, in addition to immune responses in the nasal mucosa. Studying how strictly limiting the exposure of NDV-PFS to the upper airway, possibly through lower inoculation volumes, will be critical in future studies to determine whether upper airway exposure to the vaccine provides similar levels of protection in the lungs, as this would be the case with human mucosal or nasal vaccinations.

Following the BA.2 virus challenge, we did not see increased IgA in the lungs of infected mice. We also did not see a boost effect with the BA.2 neutralization that we did in hamsters. We did not measure the presence of other antibody isotypes such as IgG or IgM in the lungs, therefore it is possible that these antibodies played a role in mediating protection. However, it is also likely that in these models, cell-mediated memory immune responses can provide protection in the airway from viral replication. Though vaccinated mice have similar lung pathology to unvaccinated mice, there appeared to be an increased infiltration of lymphocytes, which may be indicative of a cell-mediated immune response. We aimed to address this further by examining T-cell responses following vaccination. Activated OX40+ Spike-specific cytokine-producing CD4 and CD8 T cells, including poly-functional IFNγ and TNFα-producing cells, were induced following intranasal vaccination in both the lungs and spleen, indicating that site-specific and circulating systemic immune responses were generated (Fig. 6). While we did not examine cell surface markers to distinguish tissue-resident cells, the presence of spike-specific memory T cells in the lung indicates that these cells can mediate protection following challenge. Given the strong correlation of T cell polyfunctionality, improved proliferation and cytotoxicity with vaccine efficacy, identification of polyfunctional CD4 and CD8 T cell subsets following vaccination is a strong indicator of the immunogenicity of this vaccination approach^51^. Interestingly, most animals that received only a single vaccine dose did not have spike-specific T cell levels at those seen in the prime-boost animals. A single vaccine dose still provided complete protection against the lethal delta challenge, with only two animals euthanized on day three having detectable viral titers in the lungs. Even with replication in the lungs, vaccination likely prevented the virus from entering the brain to cause lethal encephalitis. We also saw breakthrough viral replication in the tissues of the single-dose mice challenged with BA.2 that was not seen in prime-boost animals, as has been observed worldwide following mRNA vaccination. This could be attributed to the evolution of the spike protein in SARS-CoV-2 variants, where modifications in regions heavily targeted by anti-spike antibodies render antibodies ineffective^71–74^. However, because of cell-mediated immune responses, to which the recognized epitopes are mostly conserved between variants, the course of infection was likely limited, as seen in the instances of breakthrough infections following mRNA vaccination^75–78^. Continuously updating the spike protein within the viral vaccine vector would be laborious, and it may only prevent breakthrough infection until a novel VOC with additional immune escape variants evolves. It is possible that lingering innate immune activation could play a role in protection, however we previously showed that control vaccination with an NDV expressing an irrelevant protein (GFP) did not provide any level of protection in Syrian hamsters^28^. Thus protection that is mediated via vaccination is antigen-specific. Therefore, it is likely that to see substantial protection against Omicron and its sub-lineages, at least two doses of NDV-PFS will be required. Further mechanistic insights into protection in mouse models could be ascertained by adoptive transfer experiments to identify cell populations that are important for protection.

Our data confirms that vaccination with ancestral strain spike protein expressed by NDV in a pre-fusion conformation can provide robust protection against SARS-CoV-2 variants, including BA.2. A previous study in mice transduced with Ad-hACE2 that evaluated an inactivated IM administered trivalent NDV-COVID-19 vaccine comprised of viruses expressing spike proteins from ancestral, Beta and Delta SARS-CoV-2 demonstrated protection against mismatched, phylogenetically distant variants, and cross-neutralizing antibodies against the Omicron variant circulating at the time of publication. Similar to our study, the monovalent NDV vaccine expressing ancestral prefusion stabilized spike induced little to no neutralizing activity against Omicron; however, the efficacy of the NDV-ancestral vaccine against Omicron challenge was not evaluated^79^. It is possible that mucosal vaccination with a replication-competent NDV-COVID-19 can induce immune responses sufficient for protection at the mucosa, while systemic vaccination with the same antigen may not provide the same level of protection against variants of concern. A combination of vaccine route and vector could have significant impacts on infection outcome, and further experiments examining the mechanism of protection are warranted. In addition, we have only tested our current vaccine formulation against BA.2, and at the time of writing, BA.2 sublineages or BA.4/5 and their sublineages, as well as recombinant Omicron lineages are the predominant circulating SARS-CoV-2 variants globally. Additional studies addressing efficacy against these viruses are needed.

One concern is that several months after vaccination, in the wake of newly emerging variants and the continuing evolution of SARS-CoV-2, antibody waning and reduced vaccine effectiveness against infection and mild disease became evident^80,81^. Our data indicate that NDV-PFS intranasal vaccination induced durable immunity that was detectable 5–6 months post-booster administration and provided significant protection against challenges with Delta and Omicron. This could alleviate some worry that recent immunization with a given vaccine followed by the emergence of a new, antibody-escaping variant will lead to reduced vaccine effectiveness, which has been addressed to some degree using updated mRNA vaccines^82^. However, their effectiveness is controversial and the rate at which they can be brought to the population does not always match the generation of novel VOCs, still resulting in breakthrough infections^82,83^. The durable protection seen in hamsters out to six months also provides some evidence that a mucosal booster could provide seasonal protection. Further studies examining longer-term efficacy in this model and others can give us a better indication of the length of protection afforded by NDV-PFS intranasal vaccination. With the rollout of bivalent variant-specific COVID-19 boosters, it is likely that future vaccines developed for SARS-CoV-2 will be specific for certain anticipated circulating variants in a manner similar to what is done with influenza vaccination. Recent updates to both Pfizer/BioNTech and Moderna’s mRNA vaaccines and to NovaVax’s protein-based vaccine to include Omicron spike proteins occurred on the order of months. The adaptability of the NDV system, which similarly involves the generation of a recombinant DNA sequence, can allow for the generation of new vaccines relatively easily. The timeline for such an update would likely be similar to that seen for the generation of seasonal influenza vaccines, which are also mass-produced in eggs.

Here we have described a newly generated chimeric NDV-vectored vaccine expressing pre-fusion stabilized SARS-CoV-2 spike protein. When administered intranasally, NDV-PFS provides protection against disease development and viral replication in Delta and Omicron-infected hamsters and mice. Our data showing protection in two distinct animal models add to the body of literature showing that NDV is a reliable and suitable vaccine vector, particularly with a goal of mucosal immunization. We also show that mucosal vaccination provided durable, long-lasting protection out to at least six months. Overall, NDV-PFS represents a highly efficacious vaccine candidate against SARS-CoV-2 and is a platform that should continue to be developed as a potential means of providing mucosal immune protection against not only SARS-CoV-2 but other respiratory viruses.

## Methods

### Engineering and propagation of NDV encoding the pre-fusion stabilized spike protein of SARS-CoV-2

An RNA and human codon-optimized cDNA encoding the SARS-CoV-2 spike protein containing the six HexaPro proline mutations^45^, an ablated furin cleavage site (RRAR to GSAS) and the transmembrane and cytoplasmic tail domain of the NDV fusion protein (amino acids 501–554 of NDV F replaced amino acids 1209–1274 of Spike) was synthesized by Genscript. The gene for this chimeric pre-fusion stabilized Spike protein (PFS) was cloned into the XbaI and MluI restriction sites of a synthetic cDNA molecular clone of lentogenic Newcastle disease virus (NDV) (based on GenBank accession number AF077761.1) harboring a leucine to alanine mutation at position 289 of the fusion protein (L289A)^84^. Following the recommended protocol for InFusion^TM^ cloning, NDV cDNA was linearized with XbaI and MluI restriction enzymes and the human codon-optimized chimeric PFS spike described above was PCR amplified (5’-GCACCGAGTTCCCCCTCTAGATTAGAAAAAATACGGGTAGAACCGCC-3’, 5’-GTTGGACCTTGGGTACGCGTTCACATCTTAGTTGTAGCCCGC-3’) and inserted between NDV P and M genes. Recombinant NDV expressing PFS spike (NDV-PFS) was rescued in Hep2 cells as described previously and subsequently amplified in specified pathogen-free embryonated chicken eggs (Canadian Food Inspection Agency), purified by depth filtration, tangential flow filtration, and iodixanol gradient ultracentrifugation before storage in 5% Sucrose at −80 °C as described previously^40,78^. Virus stocks were titrated using a TCID50 immunofluorescence assay, and the genome integrity was confirmed by RT-PCR and Sanger sequencing as described^85^.

### Cells and viruses

Vero cells (ATCC) were cultured in minimal essential medium (MEM) (Hyclone) supplemented with 5% bovine growth serum (BGS) supplemented calf (Hyclone) l-glutamine. VeroE6-TMPRSS2 cells were grown in MEM supplemented with 5% BGS, 1% non-essential amino acids (Gibco), 1 mM Na pyruvate (Gibco), 1% Penicillin/Streptomycin (Gibco) and 3 μg/ml of Puromycin (Invivogen). Cells were cultured at 37 °C with 5% CO_2_. Ancestral SARS-CoV-2 (hCoV-19/Canada/ON-VIDO-01/2020) and SARS-CoV variants of concern (Delta; hCoV-19/Canada/ON-NML-63169/2021 (B.1.617.2) and Omicron (hCoV-19/Canada/UN-295389-p1/2022; GISAID EPI_ISL_13227179; BA.2) were isolated from positive patient samples and stocks of the virus were grown in either VeroE6 cells (VIDO-01 or Delta) or VeroE6-TMPRSS2 cells (Omicron). Virus stocks were titered by TCID_50_ assay before being used for subsequent in vivo experiments.

Hep-2 (ATCC CCL-23), DF-1 (ATCC CRL-12203) and BHK-21 (ATCC CCL-10) cells were cultured at 37 °C, 5% CO_2_ in Dulbecco’s modified eagle medium (DMEM) (Cytiva) containing 5% fetal bovine serum (FBS) (Cytiva), 1% penicillin/streptomycin (Cytiva) and 1% l-glutamine (Cytiva). Modified vaccinia Ankara encoding the T7 Polymerase (MVA-T7), a kind gift from Dr. Bernard Moss, was used in the rescue of NDV-PFS. MVA-T7 was propagated and titrated in BHK-21 cells.

### Characterization of NDV-PFS by sodium dodecyl-sulfate polyacrylamide gel electrophoresis and Western blot

For the generation of infected lysates, 1 × 10^6^ DF-1 cells seeded in a six-well plate in DMEM containing 2% FBS and 125 µg/mL trypsin were infected at an MOI of 1 for 24 h. Cells were collected using a cell scraper and washed in phosphate-buffered saline (PBS) before resuspension in RIPA buffer (50 mM Tris–HCl pH 7.5, 150 mM NaCl, 1% Triton X-100, 0.1% SDS, 10 mM EDTA, 1% sodium deoxycholate) containing 1X protease inhibitor (Thermo Fisher, 87785). Cells were incubated on ice for 20 min, then centrifuged at 10,000×*g* for 15 min, and supernatants were stored at −20 °C.

Gradient gels were prepared using 6% and 10% acrylamide solutions. Briefly, 5 mL of 6% acrylamide was taken up in a 10 mL pipette, followed by 5 mL of 10% acrylamide solution. A single air bubble was introduced to mix the solution, which was then used to cast the gradient gel. 5 × 10^4^ PFU of purified NDV-PFS or NDV-GFP and 50 µg of NDV-PFS or NDV-GFP infected cell lysate was combined with 4× reducing buffer (400 mM Tris pH 6.8, 8% W/V SDS, 40% glycerol, 0.4% bromophenol blue, 400 mM β-Mercaptoethanol) and boiled at 95 °C for 10 min. Samples were resolved at 50 V for 30 min followed by 45 min at 120 V in running buffer (2.5 mM Tris, 19.2 mM Glycine, 0.01% SDS) using a BioRad Mini PROTEAN Tetra cell apparatus. SDS–PAGE gels were transferred to 0.2 µM polyvinylidene difluoride (PVDF) overnight at 4 °C in transfer buffer (24 mM Tris, 19.2 mM glycine, 20% methanol). PVDF was blocked in 5% skim milk 0.1% PBS-Tween20 (PBST; blocking buffer) for 1 h at room temperature before a 1 h incubation in either rabbit anti-SARS-CoV-2 S1 subunit (Thermo Fisher, PA5-81795) or mouse anti-NDV ribonucleoprotein (Novus Biologicals, NBP2-11633) diluted in blocking buffer. PVDF was washed 5 times in 0.1% PBST and then incubated for 1 h at room temperature in horseradish peroxidase (HRP) conjugated secondary antibody (goat anti-rabbit, Invitrogen; G21234 or goat anti-mouse IgG; Invitrogen, G21040) diluted in blocking buffer. PVDF membrane was washed 5 times in wash buffer before incubation in SuperSignalTM West Pico PLUS Chemiluminescent substrate (Thermo Fisher, 34580) for 5 min. PVDF was visualized using a BioRad ChemiDoc MP Imaging system and BioRad Image Lab 6.0.1 software. All blots shown are original scans. Images of uncropped, raw blots are included within the supplementary materials (Supplementary Fig. 10).

### Study design for animal infections

Four-six-week-old (80–100 g) or 3–4-month-old Syrian golden hamsters (*Mesocricetus auratus*) were purchased from Charles River Laboratories. Four-six-week-old human ACE2 transgenic K18 mice (B6.Cg-Tg(K18-ACE2)2Prlmn/J) were purchased from Jackson Laboratories. All animals were acclimated for at least one week before the beginning of all experiments. Hamsters were housed five per cage, and mice were housed six per cage, both separated by sex. Experimenters were not blinded to the experimental groups due to the few trained staff for rodent care in high containment. For hamster experiments, all vaccine groups consisted of 10 animals, four of which were monitored daily for weight loss and clinical signs, and six of which were euthanized on day 5 post-infection to examine viral titers. For mouse experiments, all groups consisted of 10 animals. For Delta infections, six animals were monitored for weight loss and survival, while four were euthanized on day three post-infection for examination of viral titers. For BA.2 infection, five animals from each group were euthanized on days 3 and 5 post-infection. In the 6-month challenge experiment in hamsters, two animals died during the study and were not included in the final challenge experiment. In the mouse study, four animals died during procedures, resulting in survival groups of five animals for both vaccine groups after Delta infection and two groups of four animals for tissue analysis following BA.2 challenge. All groups consisted of equal numbers of male and female animals.

Each vaccination experiment consisted of prime-only and prime-boost groups as well as a group that received PBS as a control, except for the long-term hamster challenge study, which only included prime-boosted animals and control animals. All animals were anaesthetized with inhalation isoflurane followed by immunization via intranasal instillation. NDV-PFS vaccine was administered into the nares of each animal in 100 µL (hamsters) or 50 µL (mice) of plain media. Hamsters received 10^7^ PFU of NDV-PFS, and mice received 10^6^ PFU of NDV-PFS for each vaccine dose. These doses were chosen based on our previous studies as well as others^28,29,86^. Following the initial dose of vaccine, hamsters were bled via jugular vein bleed for serum on day 21 post-vaccination and boosted with NDV-PFS on day 28. This was followed by a second bleed for serum on day 49 (day 21 post-boost) post-vaccination. Mice were vaccinated and then bled via jugular vein bleed and boosted on day 28 post-vaccination. Before the challenge, mice were again bled on day 28 post-boost. For SARS-CoV-2 challenge animals were inoculated intranasally via the same method as above with 10^5^ TCID_50_ of either SARS-CoV-2 Delta or Omicron (BA.2) in 100 µL (hamsters) or 50 µL (mice) of plain media. Mice were given food and water ad libitum throughout all experiments and were weighed and monitored daily following infection. For euthanasia, all animals were anesthetized with inhalation isoflurane and euthanized via cervical dislocation. For mouse studies, animals meeting the humane endpoint criteria were euthanized via the same method.

For the examination of cell-mediated immune responses in K18 mice following NDV-PFS vaccination, three groups of six mice (three male, three female) were used. One group was unvaccinated, another received a single intranasal NDV-PFS dose as above, and the last received two doses 54 days apart. Necropsies and tissue harvesting were performed on day 10 post-vaccination in the single-dose animals or day 5 post-boost in the two-dose animals. Tissue processing and downstream experimental procedures are described below.

### Animal ethics statement

The animal experiments described were carried out at either the National Microbiology Laboratory (NML) of the Public Health Agency of Canada or the University of Guelph. All experiments were approved by the Animal Care Committee at the Canadian Science Center for Human and Animal Health or the Institutional Animal Care Committee at the University of Guelph per guidelines from the Canadian Council on Animal Care (CCAC). The University of Guelph experiments involving mice were under animal utilization protocol #4664. The experiments at the NML were done under animal user documents H-20-010 for hamsters and H-20-011 for mice. All procedures, including vaccinations and collections, were performed under anesthesia, and all efforts were made to minimize animal suffering and reduce the number of animals used. All procedures were performed under inhalation anesthesia using isoflurane. All efforts were made to minimize animal suffering and to reduce the number of animals used. All SARS-CoV-2 infectious work was performed under biosafety level 4 (BSL-4) conditions at the National Microbiology Laboratory.

### Quantification of viral titers by TCID_50_

For the measurement of viral titers in the tissues of infected animals, TCID_50_ assays were performed. Following the necropsy, tissue samples were frozen at −80 °C. For infectious assays, titering of SARS-CoV-2 Delta was performed using Vero cells cultured in MEM. For BA.2, samples were cultured using VeroE6-TMPRSS2 cells in DMEM, as described above. Samples were thawed and placed in media supplemented with 1× l-glutamine and 1% FBS and were homogenized with 5 mm stainless steel beads in a Bead Ruptor Elite Tissue Homogenizer (Omni). Homogenates were clarified by centrifugation at 1500×*g* for 10 min, and ten-fold serial dilutions of tissue homogenates were made in the same media. Dilutions were added to 90–100% confluent cells in triplicate wells, and the cytopathic effect was read on either 4 (BA.2) or 5 dpi (Delta). TCID_50_ values per gram of tissue were calculated using the Reed and Muench method.

### Serology

For binding titers, 96-well enzyme-linked immunosorbent assay (ELISA) low binding plates (Nunc F-96 wells plate, non-treated surface, Thermo Fisher Scientific) were coated overnight with ancestral SARS-CoV-2 spike protein in PBS (Baculovirus expressed soluble spike protein made in house) at 400 ng per well. The following day, plates coated with purified recombinant protein were blocked with a blocking buffer (PBS containing 5% skim milk powder) at room temperature for 1 h. Sera were diluted 1:100 and then serially diluted two-fold in blocking buffer. Each sample was then added to ELISA plates for 1 h at 37 °C. After extensive washing with PBS-T (PBS + 0.1% Tween 20), plates were further incubated with the appropriate secondary antibody (goat-anti hamster IgG secondary antibody at 1:2000 (KPL; KP-5220-0371) or goat-anti mouse IgG secondary antibody at 1:2000 (KPL; KP-5220-0460)) for 1 h at 37 °C. Plates were then washed five times with PBS-T, and 100 µl of TMB substrate (Thermo Fisher Scientific, N301) was added into each well. After 15 min of incubation, the plate reaction was stopped by adding 100 µl of 1 M H_2_SO_4_ and analyzed on a Synergy (BioTek) absorbance microplate reader at 450 nm wavelength. Samples were considered positive, with a mean absorbance greater than the mean absorbance of the wells containing negative sera plus three standard deviations.

For the quantification of neutralizing antibodies, a microneutralization assay was performed. Briefly, 96-well tissue culture plates were plated with nearly confluent Vero (VIDO-01 or Delta) or VeroE6-TMPRSS2 (BA.2) cells. Two-fold serial dilutions of serum were made, and then each dilution was incubated for 1 h at 37 °C with 50 PFU of either SARS-CoV-2 VIDO-01, Delta or Omicron (BA.2). After incubation, media was removed from the cells, and each serum–virus mixture was added to the 96-well plates in triplicate. Plates were incubated for 4–5 days at 37 °C + 5% CO_2_, and then the cytopathic effect was examined. Neutralization titers were calculated using the Reed and Muench method and are reported as the 50% neutralization titer, described here as the dilution at which a 50% reduction in CPE was observed in the assay.

### Evaluation of cell-mediated immune responses

Male and female C57BL/6 transgenic mice expressing human angiotensin-converting enzyme II (ACE2) under the human keratin 18 promoter (B6.Cg-Tg(K18-ACE2)2Prlmn/J (K18-hACE2), Jackson Laboratory, strain code 034860) received either PBS, one or two doses of 1 × 10^6^ PFU NDV-PFS intranasally as a prime or prime-boost vaccination. All mice were euthanized on the same day, with prime-only mice euthanized 10 days after vaccination and prime-boost mice euthanized 5 days post-boost, which was administered 54 days after the prime.

Spleens and lungs were harvested for processing and immune cell isolation. Spleens were transferred into Petri dishes with Hanks balanced salt solution (HBSS) (Cytiva, SH30588.02). Using the stopper of a 5 mL syringe, spleens were pressed into a single-cell suspension. The cell suspension was filtered using a 100 µM cell strainer (Thermo Fisher, 22-363-549) prior to the addition of ACK lysis buffer [8.29 g NH_4_Cl (0.15 M), 1 g KHCO_3_ (10.0 mM), 37.2 mg Na2EDTA (0.1 mM) in 1 L MilliQ H_2_O] for removal of red blood cells. Samples were washed with HBSS and then resuspended in RPMI containing 10% FBS and 0.01% β-mercaptoethanol (cRPMI) prior to counting and seeding into 96-well round bottom plates (Greiner Bio-One, 650180). Lungs were perfused with 5 mL of PBS to remove red blood cells and weighed before enzymatic digestion with collagenase IV (1 mg/mL) and DNase I (5 µg/mL) in HBSS in a GentleMacsTM tube (Miltenyi Biotec, 130-093-237). Samples were dissociated with a GentleMacsTM tissue dissociator (Miltenyi Biotec, 130-093-235) using lung protocol A then incubated for 20 min at 37 °C before dissociating again using lung protocol B. Enzymatic digestion was neutralized by addition of 10 mL HBSS and samples were filtered through a 100 µM then 40 µM cell strainer (Thermo Fisher, 22-363-547) into a 50 mL conical tube. Cells were washed with HBSS and then subjected to ACK lysis for removal of remaining red blood cells. Cells were washed twice with HBSS before resuspension in cRPMI and subsequent plating in a round bottom 96-well plate.

Lung and spleen samples were plated in triplicate, with lung samples being divided evenly and 1 × 10^6^ splenocytes being plated per well of a 96-well round bottom plate. Samples were stimulated with a spike peptide pool (2 µg/mL) (JPT Peptide Technologies, PM-WCPV-S), Phorbol 12-myristate 13-acetate (10 ng/mL) (PMA), and ionomycin (1500 ng/mL) or left unstimulated. Unstimulated and spike peptide stimulated samples were incubated at 37 °C, 5% CO_2_ for 15 h prior to the addition of Brefeldin A (Invitrogen, 00-4506-51) for an additional 5 h. Samples stimulated with PMA/ionomycin were incubated at 37 °C, 5% CO_2_ for 1 h prior to the addition of Brefeldin A for an additional 5 h. Following stimulation, samples were centrifuged at 500×*g* for 5 min, resuspended in Fc block (CD16/32, BioLegend, 101329, 1:200 dilution), and incubated at 4 °C for 15 min. Samples were washed with FACS buffer (PBS, 0.5% bovine serum albumin), resuspended in surface staining antibodies FITC Rat Anti-Mouse CD25 (BioLegend, 102005, 1:200 dilution), PE rat anti-mouse OX40 (BioLegend, 119409, 1:200 dilution), BV510 rat anti-mouse CD4 (BioLegend, 116025, 1:200 dilution), PE-Cy7 rat anti-mouse CD8α (BioLegend, 100721, 1:200 dilution), Pacific blue rat anti-mouse CD3 (BioLegend, 100213, 1:200 dilution) and incubated at 4 °C for 20 min. Samples were washed twice with PBS, then resuspended in Zombie NIR Fixable viability dye (FVD) (BioLegend, 423105, 1:1000 dilution) and incubated at 4 °C for 30 min. Samples were washed twice with PBS before resuspension in IC fixation buffer (BioLegend, 420801) and incubated at 4 °C for 20 min. Afterward, samples were washed twice with permeabilization buffer (BioLegend, 421002) and stained with intracellular staining antibodies PerCPCy5.5 Rat Anti-Mouse TNFα (BioLegend, 506321, 1:200 dilution), APC Rat Anti-mouse IFNγ (BioLegend 505810, 1:200 dilution) for 20 min at 4 °C. Samples were washed twice in permeabilization buffer and resuspended in FACS buffer for sample analysis using a BD FACS Canto II flow cytometer and BD FACSDiva version 8.0 software.

Lymphocytes were first identified using the forward scatter area (FSC-A) and side scatter area (SSC-A). Doublets were then removed using FSC-A versus forward scatter height (FSC-H). Dead cells were removed on the basis of FVD positivity, and immune cells responsive to treatment were defined as CD25+ and OX40+. This population was further separated into either CD3+ and CD4+ or CD3+ and CD8+ to concentrate upon CD8+ and CD4+ T cells. Identification of IFNγ+ and TNFα+ populations contributes to identifying immune cell activation status. Gating strategies are represented in Supplementary Figs. 7 and 8.

### Histopathology

The cranial and caudal lobes of the lung were extracted from each hamster on day 5 and the mouse on days 3 and 5 post-challenge with Delta and BA.2, respectively. Tissues were then fixed in 10% buffered formalin and subjected to routine paraffin embedding, sectioning, and staining with hematoxylin and eosin (HE). Lesions in hamsters and mice were evaluated by evaluating the distribution of the inflammation, as well as a nominal tally of lesions (Supplementary Tables 1 and 2). The final score for each animal was calculated as the product of the distribution score by the nominal modifiers. For mice, the distribution score was derived by counting the number of perivascular/peribronchiolar inflammatory foci to account for the interstitial inflammation that was the most common lesion; if cuffs were prominent (>4 rows of lymphocytes), then the distribution score was multiplied by 2 (range 0–8). In hamsters, distribution was assessed by semiquantitatively evaluating the percentage of affected lung tissue, ranging from 0 to 4. The nominal modifiers included 3 and 10 categories for mice and hamsters, respectively. The difference in the number of nominal lesions reflected the wider variety of changes observed in hamsters. Each nominal lesion was weighted as 1, however, *vascular changes* (i.e., endothelitis) and *alveolar exudative lesion*s (i.e., edema/fibrin in alveoli) categories were weighted as 2 (as these were interpreted as more severe lesions), while *presence of hemosiderin pigment* was weighted 0.5. The final score could span 1–18 and 0–46 for mice and hamsters, respectively.

### Statistical analyses

All results were analyzed and graphed using Prism version 9 (GraphPad Software). Where appropriate, statistical tests used to determine significance included (all two-sided) Kruskal–Wallis tests with multiple comparisons, Mann–Whitney test, Wilcoxon matched-pairs sign ranked test, or two-way ANOVA, as described in the figure legends. Exact *p* values are shown for Mann–Whitney and Kruskall–Wallis comparisons (two-tailed) using Dunn’s test for multiple comparisons. For analysis of cell-mediated immune responses to NDV-PFS vaccination, means were compared using a one-way analysis of variance and Tukey’s multiple comparisons test. Exact *p* values are not shown for analyses done by two-way ANOVA.

### Reporting summary

Further information on research design is available in the Nature Research Reporting Summary linked to this article.

### Supplementary information

Supplmentary Material

REPORTING SUMMARY

## Data Availability

All data are available upon request, and inquiries should be sent to Darwyn.Kobasa@phac-aspc.gc.ca.
