# Supplementary material for: Intranasal vaccination with an NDV-vectored SARS-CoV-2 vaccine protects against Delta and Omicron challenges
Source: NPJ Vaccines. 2024 May 23;9:90. doi: 10.1038/s41541-024-00870-8 (PMC11116387; doi:10.1038/s41541-024-00870-8)
Supplement: Supplementary file 1 — Supplmentary Material [file 41541_2024_870_MOESM1_ESM.pdf]

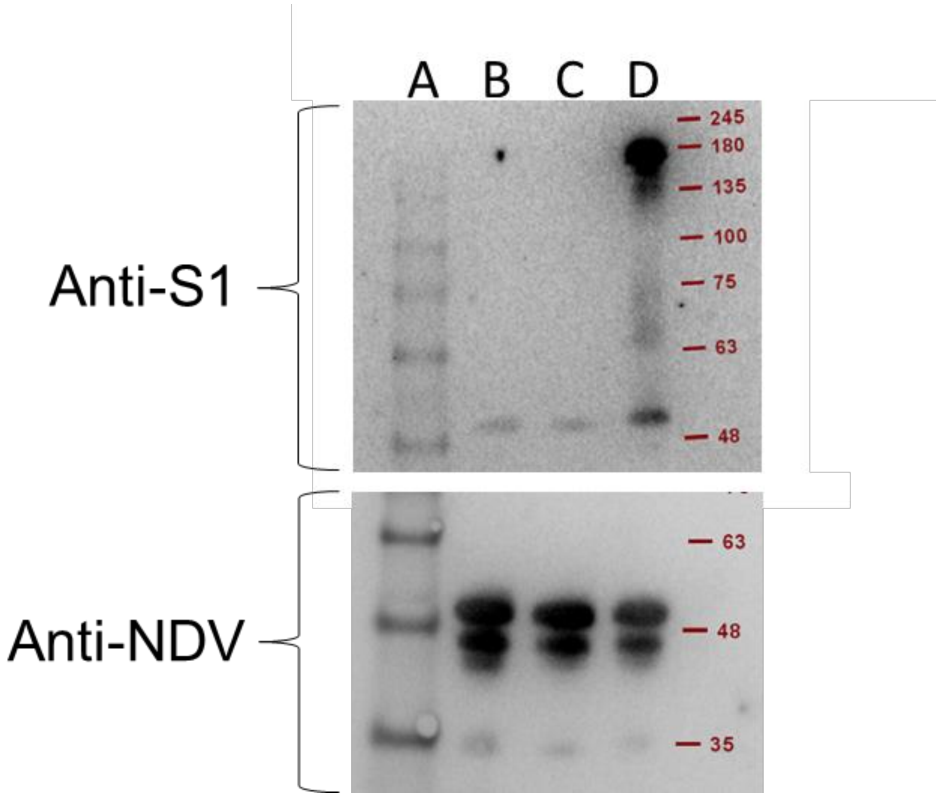

Supplementary Figure 1. A 6% to 15% SDS-PAGE gradient gel was loaded with $2x10^5$ PFU of purified NDV-GFP (B), NDV-FLS (C) or NDV-PFS (D) for comparison of spike protein expression onthe surface of NDV virions. Full length spike protein (180kDa) was detected by anti-S1antibody, with detection of NDV ribonucleoprotein complex used as a loading control.

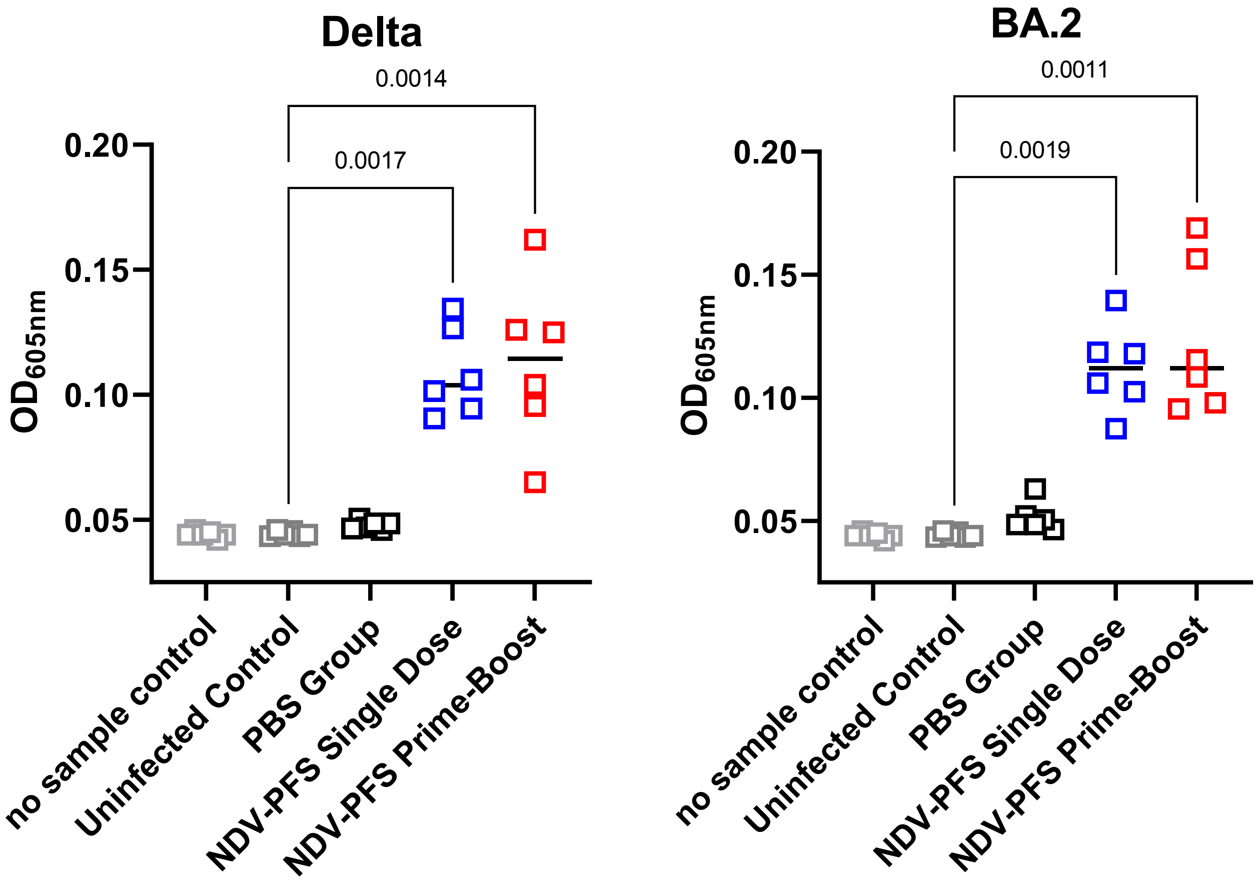

Supplementary Figure 2. IgA in hamster lung homogenates following challenge with Delta and BA.2. Significance assessed by Kruskall-Wallis test with multiple comparisons.

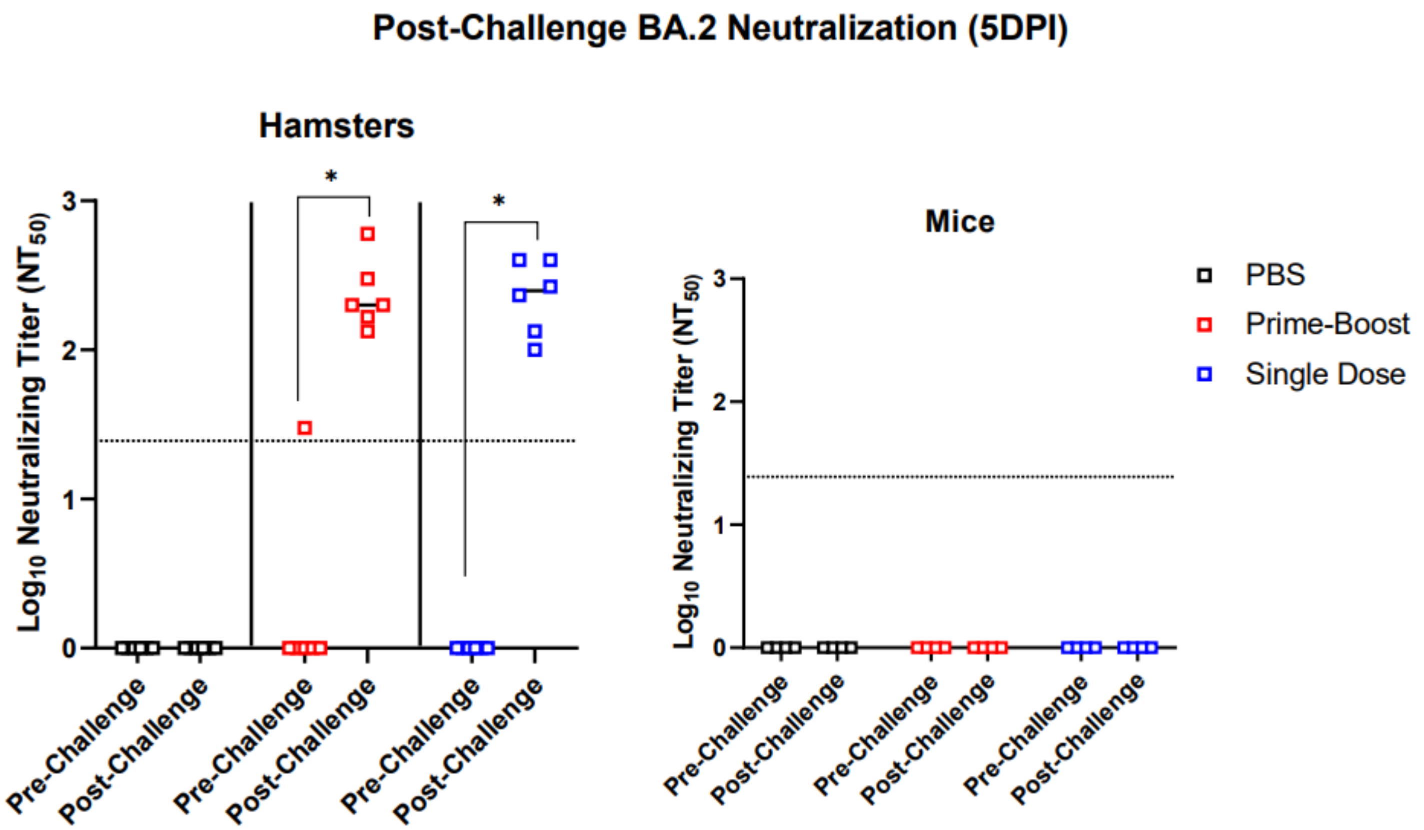

Supplementary Figure 3. BA.2 serum neutralization titers in hamsters and mice 5 days post-challenge.

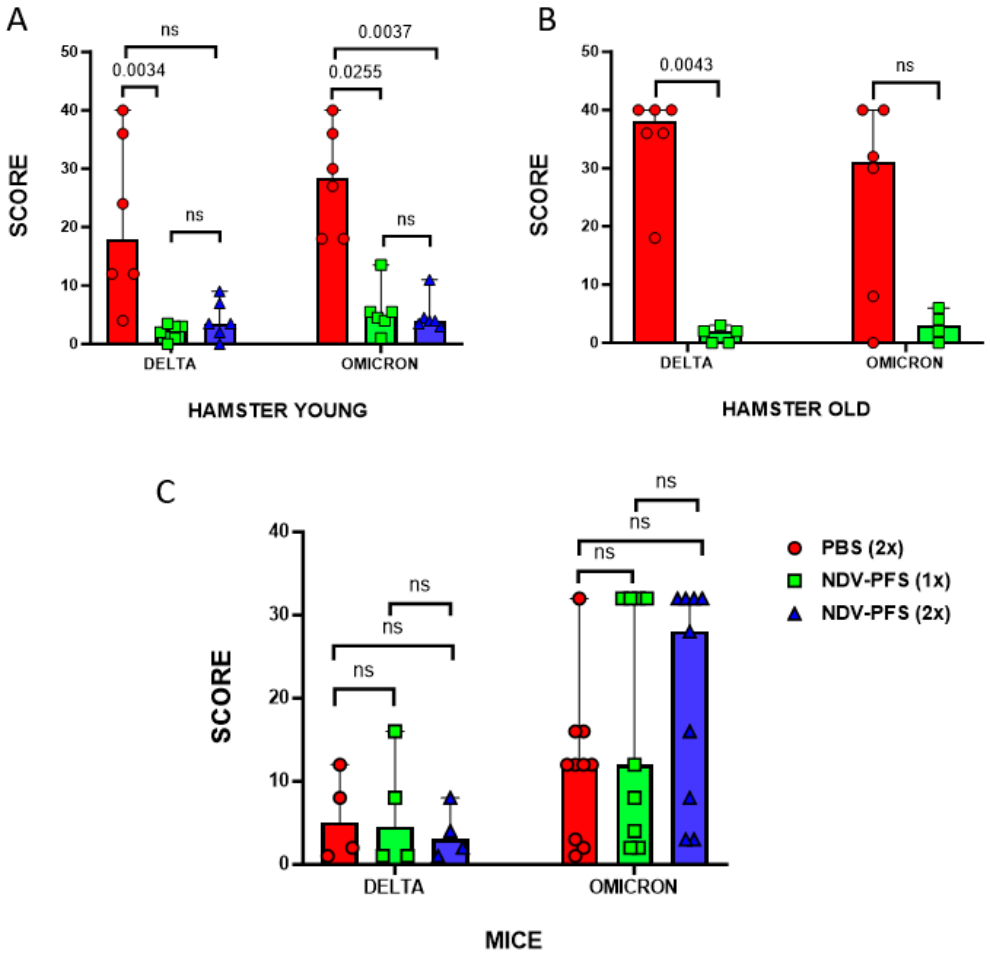

Supplementary Figure 4. Lung histopathological score in hamsters (**A**, **B**) and mice (**C**) infected with SARS-CoV-2 variants Delta and Omicron, and either vaccinated with NDV-PFS in a single (prime) or double dose (prime-boost). For figure **A** and **C**, animals were infected 28 days after vaccination, while for figure **B**, hamsters were infected 168 days after boost. Bars represent median with range and single data points. Differences between groups were tested by the Kruskal-Wallis test with Dunn's test for multiple comparisons (significance, $p < 0.05$). Vaccinated hamsters have significantly lower histopathological scores compared to the unvaccinated and infected hamsters, with no differences between variants. In mice,vaccinated and animals did not show protection from histopathological lesions. Especially in OMICRON-infected mice, a trend towards more severe histological scores was mainly driven by perivascular accumulation of lymphocytes (see Supplementary Figure 5).

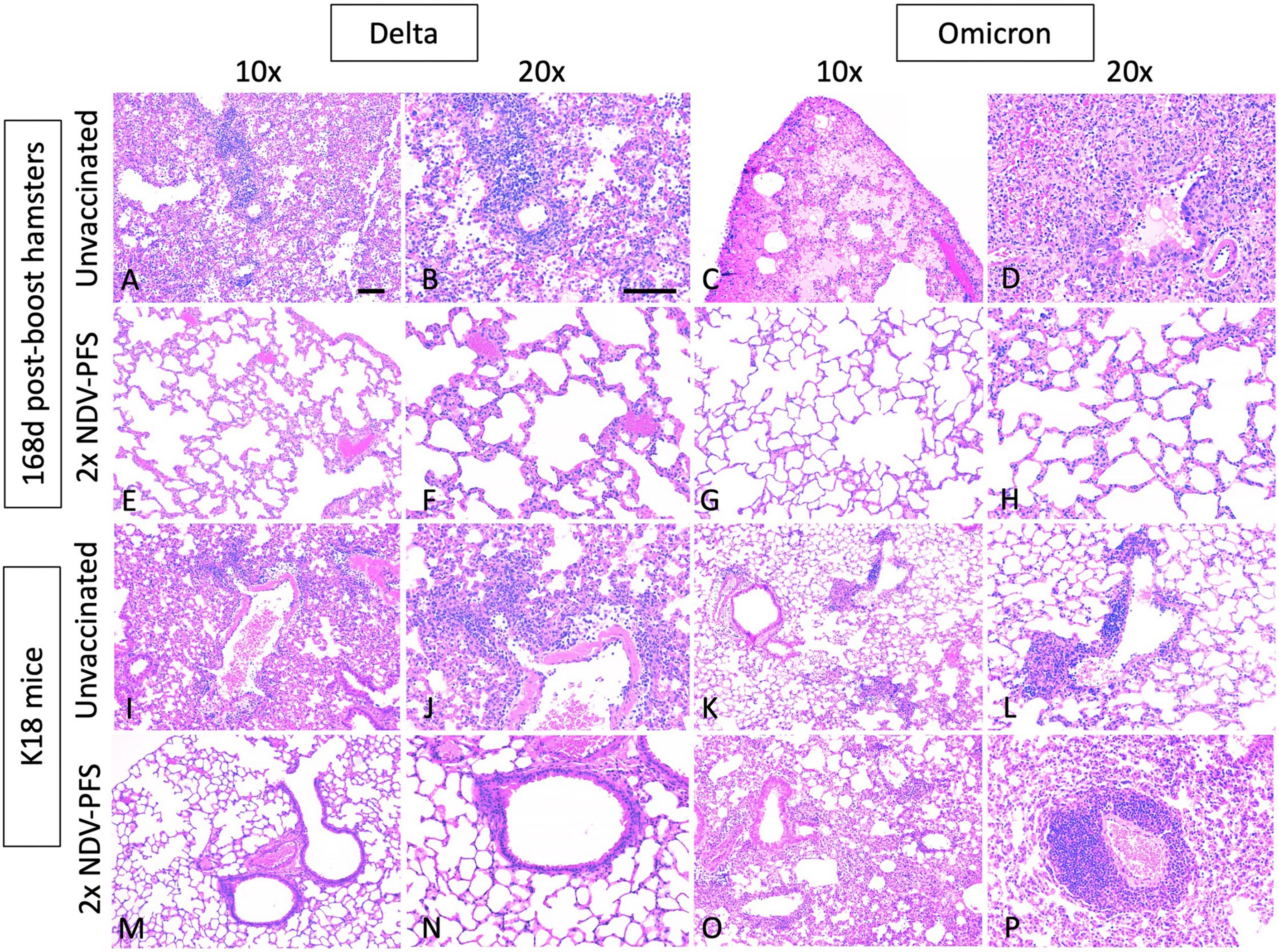

Supplementary Figure 5. Representative microscopic lesions in hamsters (rows 1 and 2) and mice (rows 3 and 4) and infected with SARS-CoV-2 Delta (panel columns 1 and 2) and Omicron (panel columns 3 and 4), and either vaccinated or not. All hamster tissues were collected D5 postchallenge. Tissues from mice challenged with Delta or BA.2 were collected at D3 and D5 post-challenge, respectively. **Hamsters**. Unvaccinated hamsters infected with Delta or Omicron variants show severe lesions,consisting of interstitial pneumonia with accumulation of lymphocytes in perivascular to interalveolar position, and accumulation of sloughed cells in the alveolar spaces (panels **A**, **B**), as well as more exudative lesions characterized by edema and fibrin admixed with inflammatory cells and red blood cells (hemorrhage) in the alveolar spaces (**C**, **D**). Hamsters challenged 186 days after vaccination with either variant show lack of inflammation and well aerated alveoli (**E**-**H**). **Mice**. Mice infected with Delta variant showed mild lesions, spanning from perivascular accumulation of lymphocytes (**I**, **J**) to lack of inflammation (**M**, **N**). In mice infected with Omicron variant, lesions were more prominent, and were mainly driven by prominent perivascular/peribronchiolar accumulation of lymphocytic cuffs (**K**-**P**). For all pictures, hematoxylin and eosin (HE) staining, with original magnification 10 X (bar = 100 μm) or 20 X (bar = 100 μm).

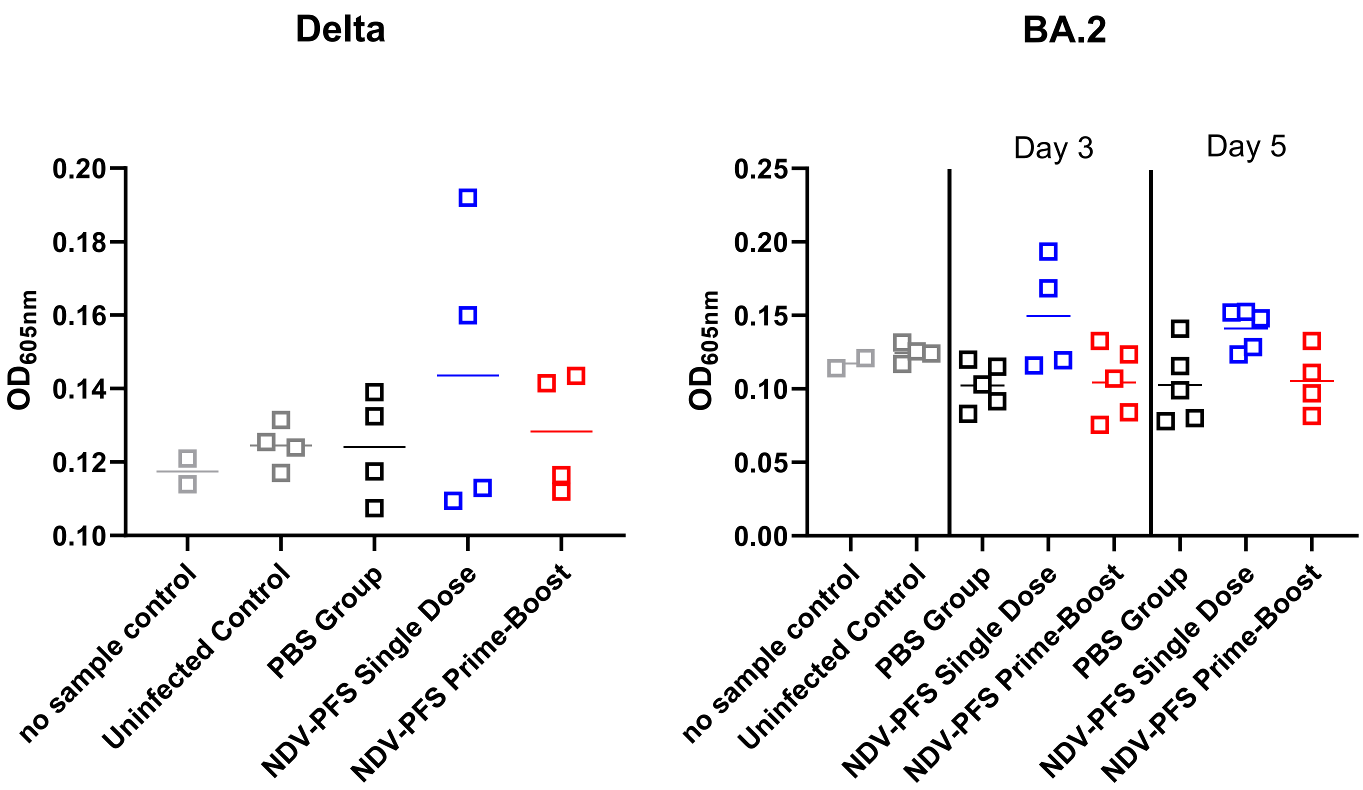

Supplementary Figure 6. IgA in mouse lung homogenates following challenge with Delta and BA.2.

Male

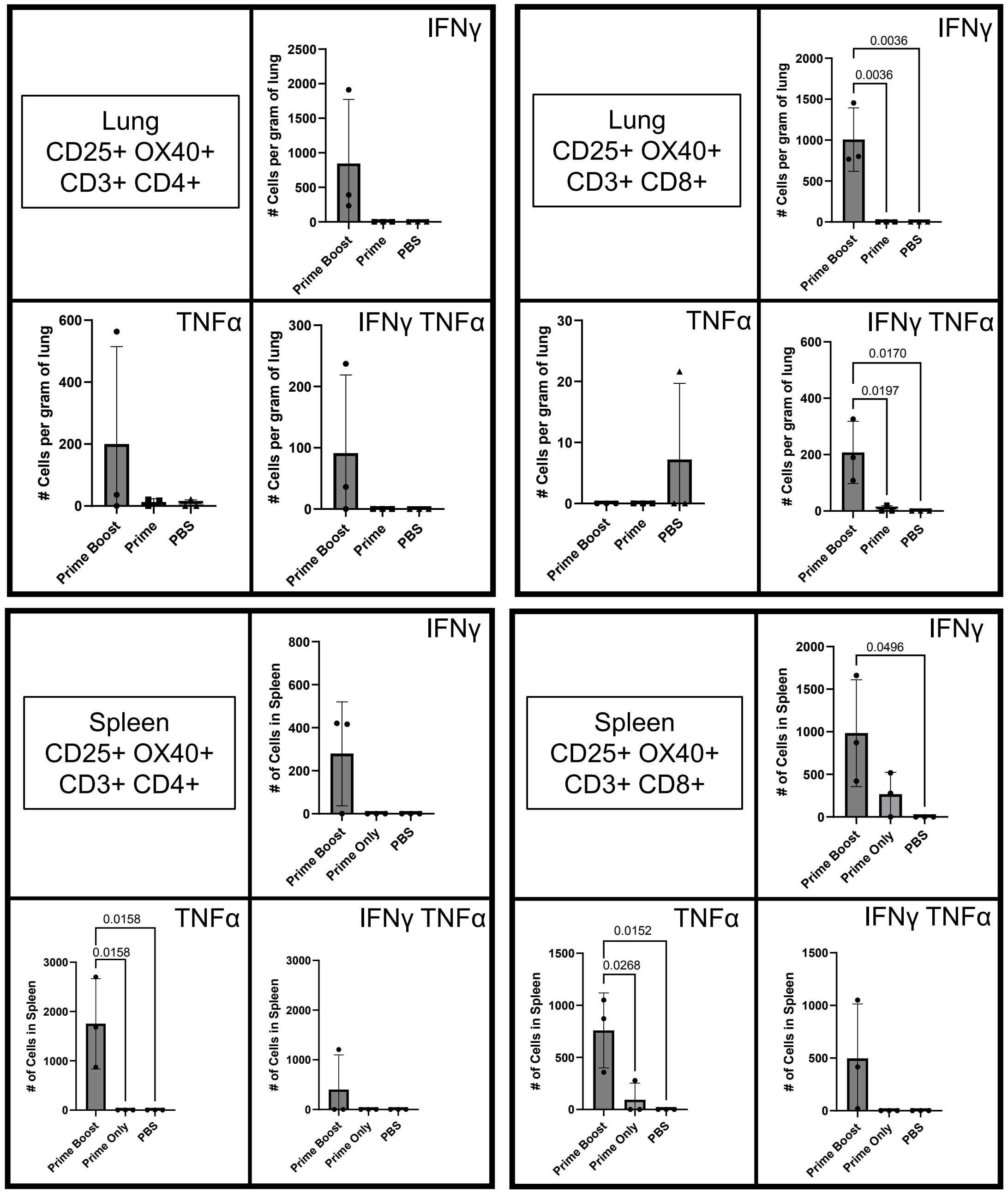

Supplementary Figure 7a. Evaluation of live activated CD4 and CD8 T cells (CD25+, OX40+,CD3+) in the lungs and spleens of male (a) and female (b) K18 mice for their production of TNFα and IFNγ. Mice received either PBS, one or two doses of $1x10^6$ PFU ofNDV-PFS intranasally. Animals receiving one dose were euthanized 10 days post-vaccination and those receiving two, 5 days after the second dose. Significance assessed by ordinary one way ANOVA, **, p< 0.005; *, p<0.05.

Female

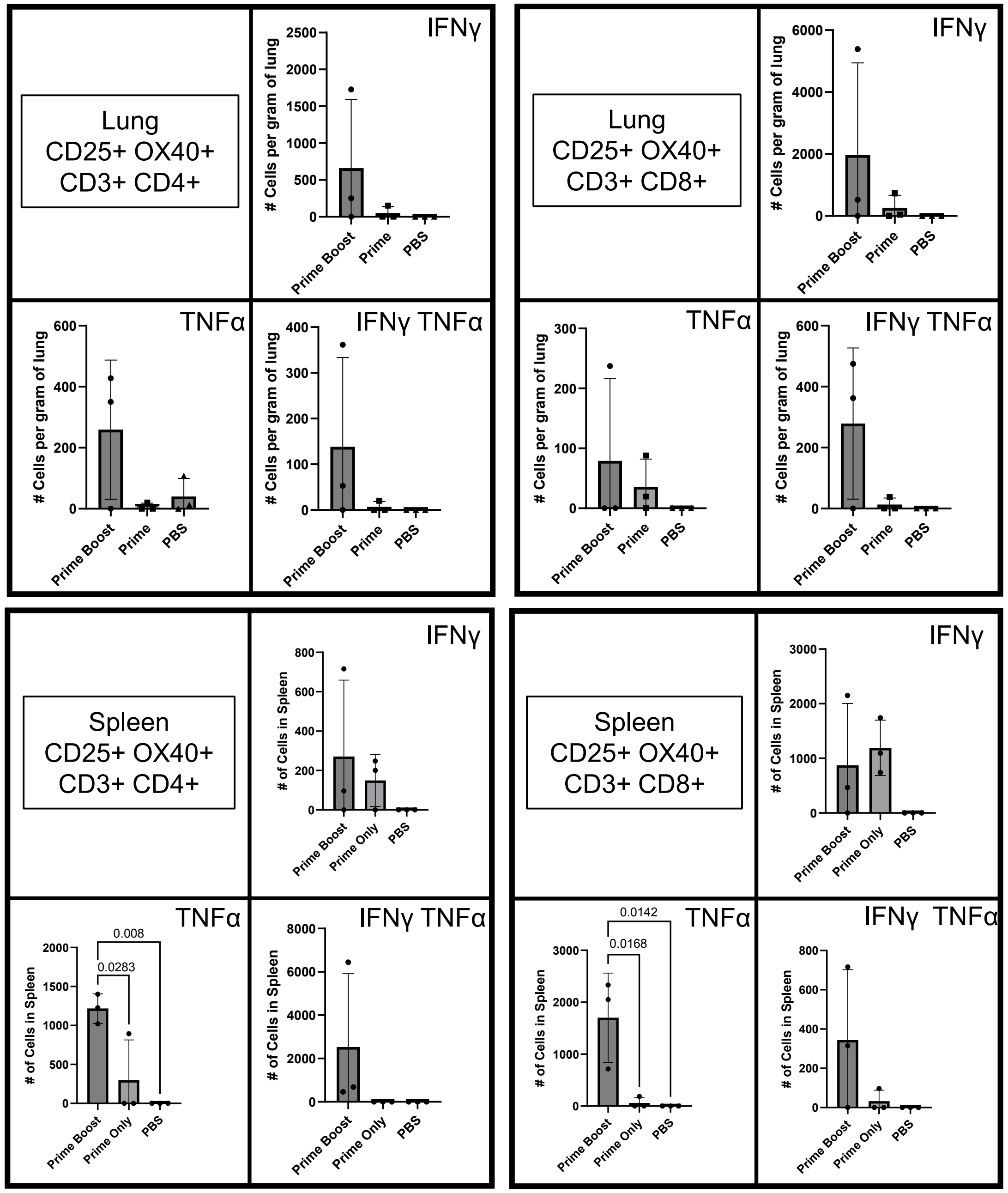

Supplementary Figure7b.

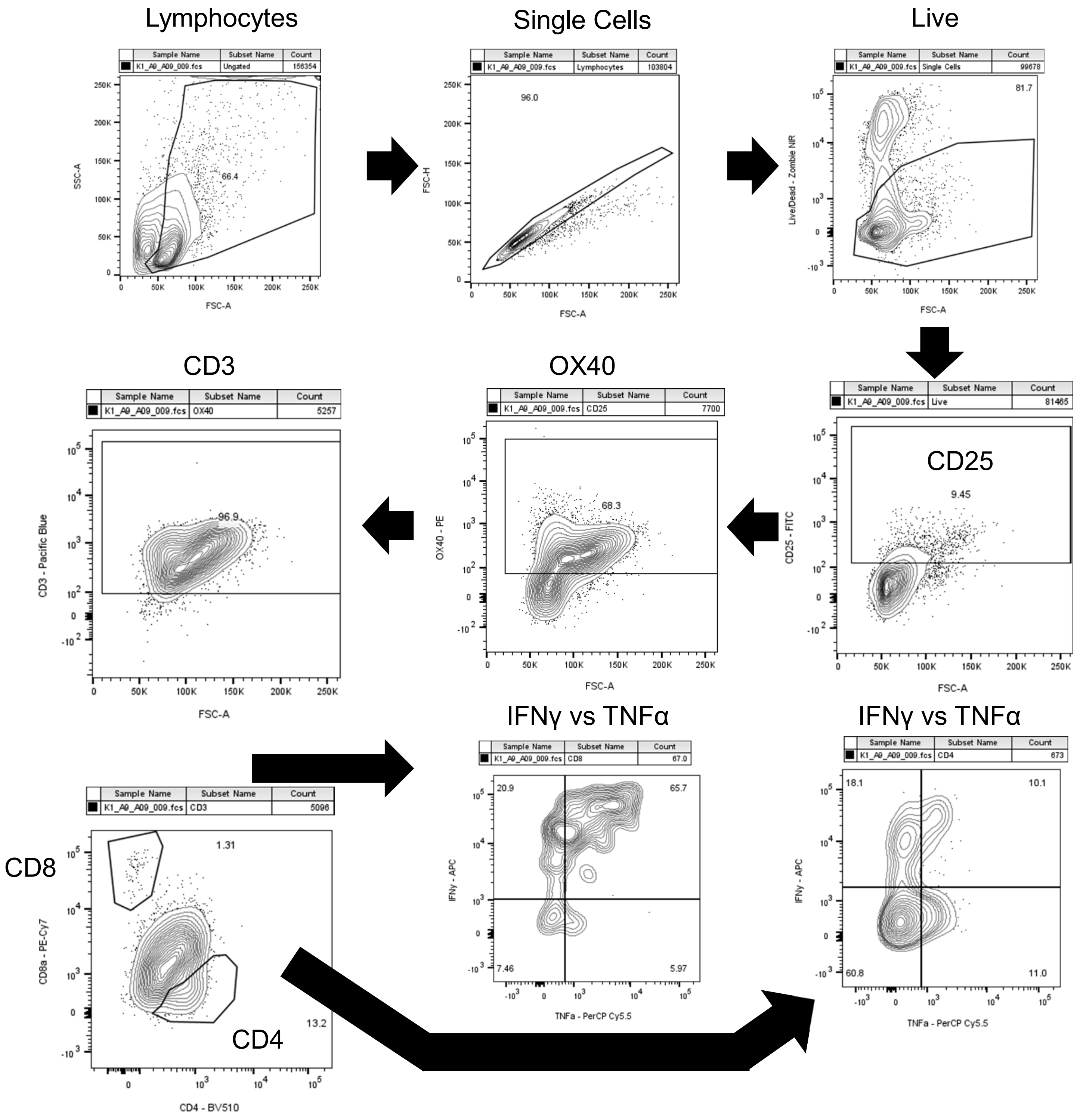

Supplementary Figure 8. Lung gating strategy.

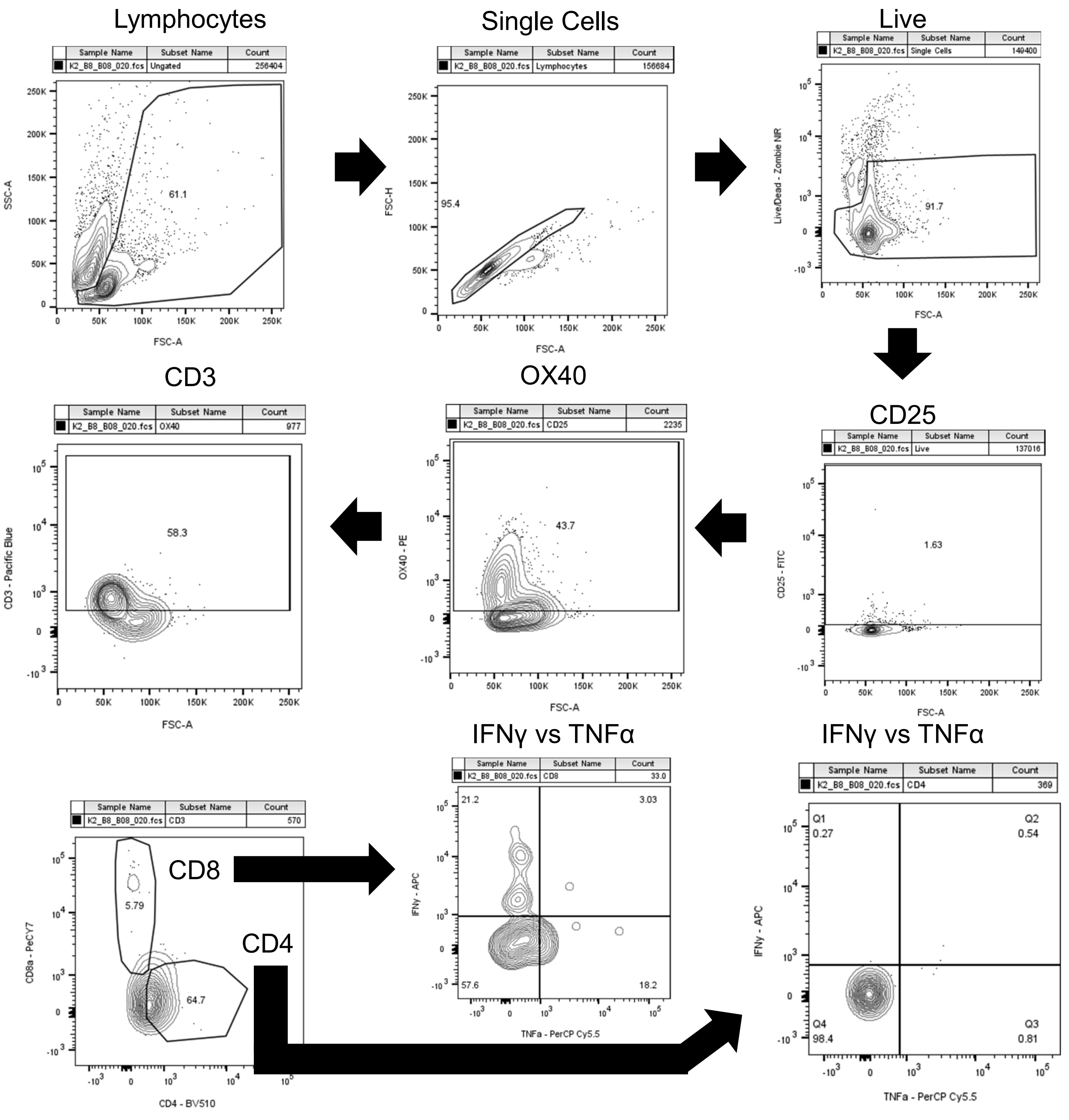

Supplementary Figure 9. Spleen gating strategy.

Figure 1 Anti-S1

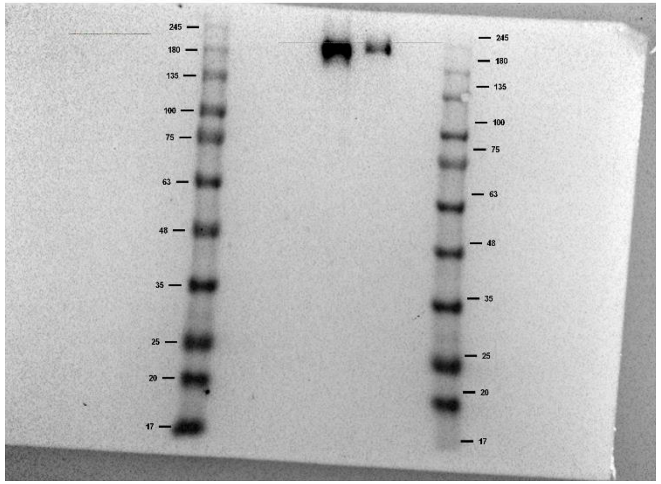

Figure 1 Anti-NDV

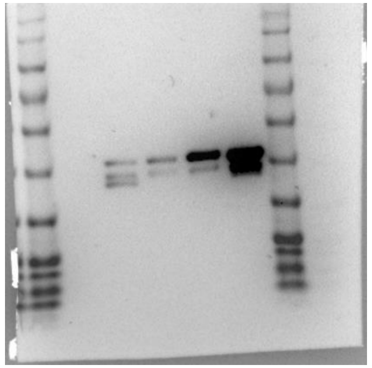

Supplementary
Figure 1 Anti-S1

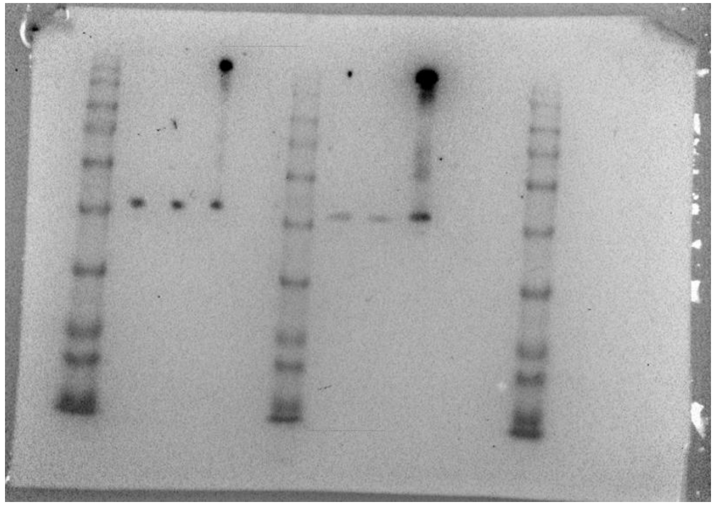

Supplementary
Figure 1 Anti-NDV

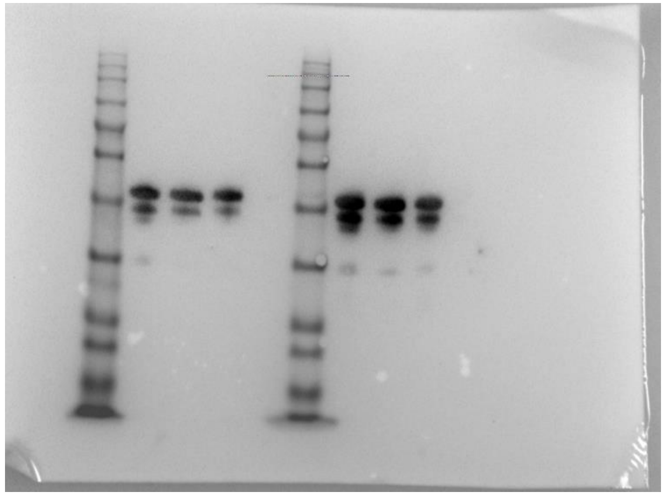

Supplementary Figure 10. Raw Western Blot Images. All images shown are uncropped, whole blots included in Figure 1 and Supplementary Figure 1.

SUPPLEMENTARY TABLE 1

Supplementary Table 1. Scoring scheme for mice.

Distribution of interstitial inflammation Lymphocytic cuffs around vessels/bronchioles				Presence of prominent cuffs		Nominal modifiers			
0-5	6-10	11-15	16-20	< 4 rows of lymphocytes	> 4 rows of lymphocytes	Alveolar exudation of cells	Endotheliitis**	Macrophages with pigment	General*
1	2	3	4	1	2	1	2	0.5	1
**FINAL SCORE**: (Distribution X cuff score) X nominal modifiers [1-36]									

*Note, a score =1 was given to all mice in the general category, as some perivascular inflammation was virtually present in each mouse, and a no true 0 scores were present. **Severe vascular changes were interpreted as presence of clusters of lymphocytes immediately below the endothelium.

SUPPLEMENTARY TABLE 2

Supplementary Table 2. Scoring scheme for hamsters.

Area (% of affected lung)					Nominal modifiers									
0-5%	5%-25%	26 -50%	51-75%	76-100%	Edema / hemorrhage around large vessels / bronchi	Alveolar edema / fibrin Hyaline membranes/	Alveolar exudation of cells	Perivascular peribronchial inflammation	Hemorrhage in alveoli	Endotheliitis**	Hyperplasia terminal bronchioles / type II pneumocytes	Hyperplasia of ciliate epithelia	Pleural activation	Macrophages with pigment
0	1	2	3	4	1	2	1	1	1	2	1	1	1	0.5
**FINAL SCORE**: Area score X nominal modifiers [0-46]														

**Severe vascular changes were interpreted as presence of clusters of lymphocytes immediately below the endothelium.
