# Supplementary material for: Intranasal vaccination with an NDV-vectored SARS-CoV-2 vaccine protects against Delta and Omicron challenges
Source: NPJ Vaccines. 2024 May 23;9:90. doi: 10.1038/s41541-024-00870-8 (PMC11116387; doi:10.1038/s41541-024-00870-8)
Supplement: Supplementary file 2 — REPORTING SUMMARY [file 41541_2024_870_MOESM2_ESM.pdf]

nature portfolio

Corresponding author(s): Darwyn Kobasa

Last updated by author(s): Mar 6, 2024

Reporting Summary

Nature Portfolio wishes to improve the reproducibility of the work that we publish. This form provides structure for consistency and transparency in reporting. For further information on Nature Portfolio policies, see our Editorial Policies and the Editorial Policy Checklist.

Statistics

For all statistical analyses, confirm that the following items are present in the figure legend, table legend, main text, or Methods section.

n/a	Confirmed	
☐	☒	The exact sample size (*n*) for each experimental group/condition, given as a discrete number and unit of measurement
☐	☒	A statement on whether measurements were taken from distinct samples or whether the same sample was measured repeatedly
☐	☒	The statistical test(s) used AND whether they are one- or two-sided<br>*Only common tests should be described solely by name; describe more complex techniques in the Methods section.*
☐	☒	A description of all covariates tested
☐	☒	A description of any assumptions or corrections, such as tests of normality and adjustment for multiple comparisons
☐	☒	A full description of the statistical parameters including central tendency (e.g. means) or other basic estimates (e.g. regression coefficient) AND variation (e.g. standard deviation) or associated estimates of uncertainty (e.g. confidence intervals)
☐	☒	For null hypothesis testing, the test statistic (e.g. *F*, *t*, *r*) with confidence intervals, effect sizes, degrees of freedom and *P* value noted<br>*Give P values as exact values whenever suitable.*
☒	☐	For Bayesian analysis, information on the choice of priors and Markov chain Monte Carlo settings
☒	☐	For hierarchical and complex designs, identification of the appropriate level for tests and full reporting of outcomes
☒	☐	Estimates of effect sizes (e.g. Cohen's *d*, Pearson's *r*), indicating how they were calculated

Our web collection on statistics for biologists contains articles on many of the points above.

Software and code

Policy information about availability of computer code

Data collection	Raw data were collected in Microsoft Excel 16.0.
Data analysis	All results were analyzed and graphed using Prism version 9 (Graphpad Software). Where appropriate, statistical tests used to determine significance included (all two-sided) Kruskal-Wallis tests with multiple comparisons, Mann-Whitney test, Wilcoxon matched-pairs sign ranked test, or two-way ANOVA, as described in the figure legends. Exact p values are shown for Mann-Whitney and Kruskall-Wallis comparisons (two-tailed) using Dunn's test for multiple comparisons. For analysis of cell-mediated immune responses to NDV-PFS vaccination, means were compared using one-way analysis of variance and Tukey's multiple comparisons test. Exact p values are not shown for analyses done by two-way ANOVA.<br>FlowJo V10.9.0 was used to analyze flow cytometry samples.

For manuscripts utilizing custom algorithms or software that are central to the research but not yet described in published literature, software must be made available to editors and reviewers. We strongly encourage code deposition in a community repository (e.g. GitHub). See the Nature Portfolio guidelines for submitting code & software for further information.

Data

Policy information about availability of data

All manuscripts must include a data availability statement. This statement should provide the following information, where applicable:

- Accession codes, unique identifiers, or web links for publicly available datasets
- A description of any restrictions on data availability
- For clinical datasets or third party data, please ensure that the statement adheres to our policy

All data are available upon request, and inquiries should be sent to Darwyn.Kobasa@phac-aspc.gc.ca

Research involving human participants, their data, or biological material

Policy information about studies with human participants or human data. See also policy information about sex, gender (identity/presentation), and sexual orientation and race, ethnicity and racism.

Reporting on sex and gender	N/A
Reporting on race, ethnicity, or other socially relevant groupings	N/A
Population characteristics	N/A
Recruitment	N/A
Ethics oversight	N/A

Note that full information on the approval of the study protocol must also be provided in the manuscript.

Field-specific reporting

Please select the one below that is the best fit for your research. If you are not sure, read the appropriate sections before making your selection.

☒ Life sciences ☐ Behavioural & social sciences ☐ Ecological, evolutionary & environmental sciences

For a reference copy of the document with all sections, see nature.com/documents/nr-reporting-summary-flat.pdf

Life sciences study design

All studies must disclose on these points even when the disclosure is negative.

Sample size	Sample sizes were calculated a priori based on estimates of expected outcome required to demonstrate vaccine efficacy and reduction in viral burden in tissues aduring prior work with related rodent species and the NDV vaccine candidates.
Data exclusions	No data was excluded from the analyses.
Replication	Animal experiments were not replicated and studies were designed with sufficient group sizes for statistical significance in evaluation of outcome.
Randomization	Animals were randomly assigned to test groups.
Blinding	Blinding of the animal experiments was not performed while experiments were being carried out in containment; however, the resulting samples were assigned numerical sample IDs, and the raw data was not linked to the experimental groups until after the data were generated and were being analyzed.

Reporting for specific materials, systems and methods

We require information from authors about some types of materials, experimental systems and methods used in many studies. Here, indicate whether each material, system or method listed is relevant to your study. If you are not sure if a list item applies to your research, read the appropriate section before selecting a response.

Materials & experimental systems

n/a	Involved in the study
☐	☒ Antibodies
☐	☒ Eukaryotic cell lines
☒	☐ Palaeontology and archaeology
☐	☒ Animals and other organisms
☒	☐ Clinical data
☒	☐ Dual use research of concern
☒	☐ Plants

Methods

n/a	Involved in the study
☒	☐ ChIP-seq
☐	☒ Flow cytometry
☒	☐ MRI-based neuroimaging

Antibodies

Antibodies used	rabbit anti-SARS-CoV-2 S1 subunit (ThermoFisher, PA5-81795; Lot Number: VG3036241); mouse anti-NDV ribonucleoprotein (Novus Biologicals, NBP2-11633); goat anti-rabbit, Invitrogen; G21234; goat anti-mouse IgG; Invitrogen, G21040; goat-anti hamster IgG secondary antibody at 1:2000 (KPL; KP-5220-0371); goat-anti mouse IgG secondary antibody at 1:2000 (KPL; KP-5220-0460); FITC Rat Anti-Mouse CD25 (BioLegend, 102005, 1:200 dilution); PE Rat Anti-Mouse OX40 (BioLegend, 119409, 1:200 dilution); BV510 Rat Anti-Mouse CD4 (BioLegend, 116025, 1:200 dilution); PE-Cy7 Rat Anti-Mouse CD8α (BioLegend, 100721, 1:200 dilution); Pacific Blue Rat Anti-Mouse CD3 (BioLegend, 100213, 1:200 dilution); PerCPCy5.5 Rat Anti-Mouse TNFα (BioLegend, 506321, 1:200 dilution); APC Rat Anti-mouse IFNγ (BioLegend 505810, 1:200 dilution)
Validation	ThermoFisher, PA5-81795, PA5-81795 has cross-reactivity in ELISA and WB with SARS-CoV-2 (2019-nCoV) Spike S1 and SARS-CoV-2 (2019-nCoV) Spike RBDPA5-81795 was prepared using the sequence encoding the S1 subunit of SARS-CoV (isolate:WH20) spike (AAX16192.1).<br>Novus Biologicals, NBP2-11633- manufacturer's website states specific to NDV RNP, validated for ELISA Flow Cytometry, Immunocytochemistry/ Immunofluorescence, Immunohistochemistry based on published studies (i.e. Warner BM, Santry LA, Leacy A Et al. Intranasal vaccination with a Newcastle disease virus-vectored vaccine protects hamsters from SARS-CoV-2 infection and disease iScience 2021-11-19 [PMID: 34632328] (ICC/IF, WB))<br>BioLegend, 102005 - manufacturer states Each lot of this antibody is quality control tested by immunofluorescent staining with flow cytometric analysis.<br>BioLegend, 119409 - manufacturer states Each lot of this antibody is quality control tested by immunofluorescent staining with flow cytometric analysis.<br>BioLegend, 116025- manufacturer states Each lot of this antibody is quality control tested by immunofluorescent staining with flow cytometric analysis.<br>BioLegend, 100721 - manufacturer states Each lot of this antibody is quality control tested by immunofluorescent staining with flow cytometric analysis.<br>BioLegend, 100213 - manufacturer states Each lot of this antibody is quality control tested by immunofluorescent staining with flow cytometric analysis.<br>BioLegend, 506321 - manufacturer states Each lot of this antibody is quality control tested by immunofluorescent staining with flow cytometric analysis.<br>BioLegend 505810 - manufacturer states Each lot of this antibody is quality control tested by immunofluorescent staining with flow cytometric analysis.

Eukaryotic cell lines

Policy information about cell lines and Sex and Gender in Research

Cell line source(s)	Vero cells (ATCC), Hep-2 (ATCC CCL-23), DF-1 (ATCC CRL-12203), BHK-21 (ATCC CCL-10); VeroE6-TMPRSS2 (BPS Bioscience).
Authentication	Vero cells (ATCC), Hep-2 (ATCC CCL-23), DF-1 (ATCC CRL-12203), BHK-21 (ATCC CCL-10) - These cell lines were authenticated by the ATCC, employing morphology, karyotyping, and PCR based approaches to confirm the identity of the cells.<br>VeroE6-TMPRSS2 - manufacturer confirms cell line is a rrecombinant clonal stable Vero E6, derived from ATCC #CRL-1586, and constitutively expressing full length human TMPRSS2
Mycoplasma contamination	Cell lines were confirmed to be negative for Mycoplasma contamination.
Commonly misidentified lines (See ICLAC register)	No commonly misidentified cell lines were used in these studies.

Animals and other research organisms

Policy information about studies involving animals; ARRIVE guidelines recommended for reporting animal research, and Sex and Gender in Research

Laboratory animals	Four-six-week-old (80-100 gram) or 3–4-month-old Syrian golden hamsters (Mesocricetus auratus) were purchased from Charles River Laboratories. Four-six-week-old human ACE2 transgenic K18 mice (B6.Cg-Tg(K18-ACE2)2Prlmn/J) were purchased from Jackson Laboratories.

Wild animals	This study did not include wild animals.
Reporting on sex	Animal groups were equally divided between males and females.
Field-collected samples	N/A
Ethics oversight	The animal experiments described were carried out at either the National Microbiology Laboratory (NML) of the Public Health Agency of Canada or the University of Guelph. All experiments were approved by the Animal Care Committee at the Canadian Science Center for Human and Animal Health or the Institutional Animal Care Committee at the University of Guelph per guidelines from the Canadian Council on Animal Care (CCAC).

Note that full information on the approval of the study protocol must also be provided in the manuscript.

Plants

Seed stocks	N/A
Novel plant genotypes	N/A
Authentication	N/A

Flow Cytometry

Plots

Confirm that:

☒ The axis labels state the marker and fluorochrome used (e.g. CD4-FITC).

☒ The axis scales are clearly visible. Include numbers along axes only for bottom left plot of group (a 'group' is an analysis of identical markers).

☒ All plots are contour plots with outliers or pseudocolor plots.

☒ A numerical value for number of cells or percentage (with statistics) is provided.

Methodology

Sample preparation	Spleens from C57/Bl6 transgenic mice (B6.Cg-Tg(K18-ACE2)2Prlmn/J) were harvested into 1.5mL tubes containing HBSS. Following transfer to a petri dish, spleens were pressed into single cell suspensions using a 5mL syringe. Cell suspension was passed through a 100 uM cell strainer prior to treatment with ACK lysis buffer to remove blood cells. Samples were washed with HBSS and resuspended in RPMI containing 10% FBS and 0.01% beta-Mercaptoethanol prior to cell counting via hemocytometer with trypan blue and plating 1,000,000 cells per well in a round bottom 96-well plate.<br><br>Lungs from C57/Bl6 transgenic mice (B6.Cg-Tg(K18-ACE2)2Prlmn/J) were perfused with 5mL of PBS to flush red blood cells and weighed prior to enzymatic digestion with collagenase IV (1 mg/mL) and DNase I (5ug/mL) in HBSS using a GentleMacs TM tube. Samples were further dissociated using a GentleMacsTM tissue dissociator on lung protocol A, incubated for 20min at 37C with a final dissociation using lung protocol B. Enzymatic digestion was neutralized by addition of 10mL HBSS and passed through a 100uM then 40uM cell strainer. Cells were washed with HBSS and subjected to ACK lysis buffer for removal of red blood cells. Cells were washed twice with HBSS prior to resuspension in cRPMI (described above) and plating in 96-well round bottom plates.<br><br>Each cell suspension was plated in triplicate, with 1,000,000 splenocytes plated per well. Samples were stimulated with 2 ug/mL of an overlapping spike peptide pool or 10 ng/mL PMA and 1500 ng/mL Ionomycin or left unstimulated for 15 hours at 37C, 5% CO2 prior to addition of Brefeldin A for an additional 5 hours at 37C, 5% CO2. PMA/Ionomycin stimulation was 1 hour prior to Brefeldin A addition and further incubation for 5 hours. After stimulation cells were pelleted and resuspended in Fc block for 15min at 4C. Samples were washed with FACS (PBS, 0.5% BSA) then resuspended in surface staining antibodies (CD25-FITC, OX40-PE, CD4-BV510, CD8a-PeCy7, CD3-PacificBlue) for 20min at 4C. Cells were washed twice in PBS then stained with Zombie NIR fixable viability dye for 30min at 4C. Following two PBS washes, cells were resuspended in IC fixation buffer for 20min at 4C, washed twice with permeabilization buffer and subjected to intracellular staining (TNFa-PerCpCy5.5, IFNy-APC) for 20min at 4C. Samples were washed twice in permeabilization buffer before resuspension in FACS buffer for analysis.
Instrument	Cells were analyzed using a BD FACS CANTO II flow cytometer.
Software	BD FACS DIVA v8.0 was used during sample acquisition. FlowJo V10.9.0 was used to analyze samples.

Cell population abundance	ACK lysis of red blood cells, numerous washes and filtration of cell suspensions were employed to promote sample purity.
Gating strategy	Lymphocytes were first identified using FSC-A vs SSC-A followed by removal of doublets using FSC-A vs FSC-H. Dead cells were removed based on Zombie NIR dye positive signal and immune cells responsive to treatment identified as first CD25+ then OX40+. This population was further separated by CD3+ signal, then expression of CD4 vs CD8. Finally, TNFa vs IFNy was used to indicate immune cell activation status.<br><br>Gates were first set by following the gating strategy above for untreated and unstimulated samples. After applying this gating strategy to all samples, PMA/Ionomycin samples were then evaluated to ensure activated populations fall into the gates set during evaluation of negative, unstimulated samples. If any modifications were required, these were then applied to all samples. Once gates were determined using negative, unstimulated and PMA/Ionomycin stimulated samples, values were exported for further analysis and graphing.

☒ Tick this box to confirm that a figure exemplifying the gating strategy is provided in the Supplementary Information.
